# Development of a CRISPR/Cpf1 system for targeted gene disruption in *Aspergillus aculeatus* TBRC 277

**DOI:** 10.1186/s12896-021-00669-8

**Published:** 2021-02-11

**Authors:** Dede Abdulrachman, Lily Eurwilaichitr, Verawat Champreda, Duriya Chantasingh, Kusol Pootanakit

**Affiliations:** 1 Institute of Molecular Biosciences, Mahidol University, Salaya, Nakhon Pathom, Thailand; 2 Thailand Bioresource Research Center (TBRC), National Center for Genetic Engineering and Biotechnology (BIOTEC), Thailand Science Park, Khlong Luang District, Pathumthani Thailand; 3 Enzyme Technology Laboratory, National Center for Genetic Engineering and Biotechnology (BIOTEC), Thailand Science Park, Khlong Luang District, Pathumthani Thailand

**Keywords:** CRISPR/Cpf1, *pyrG*, 5-FOA, FnCpf1, Gene editing, Filamentous fungi, *Aspergillus*

## Abstract

**Background:**

CRISPR-Cas genome editing technologies have revolutionized biotechnological research particularly in functional genomics and synthetic biology. As an alternative to the most studied and well-developed CRISPR/Cas9, a new class 2 (type V) CRISPR-Cas system called Cpf1 has emerged as another versatile platform for precision genome modification in a wide range of organisms including filamentous fungi.

**Results:**

In this study, we developed AMA1-based single CRISPR/Cpf1 expression vector that targets *pyrG* gene in *Aspergillus aculeatus* TBRC 277, a wild type filamentous fungus and potential enzyme-producing cell factory. The results showed that the Cpf1 codon optimized from *Francisella tularensis* subsp. *novicida* U112, FnCpf1, works efficiently to facilitate RNA-guided site-specific DNA cleavage. Specifically, we set up three different guide crRNAs targeting *pyrG* gene and demonstrated that FnCpf1 was able to induce site-specific double-strand breaks (DSBs) followed by an endogenous non-homologous end-joining (NHEJ) DNA repair pathway which caused insertions or deletions (indels) at these site-specific loci.

**Conclusions:**

The use of FnCpf1 as an alternative class II (type V) nuclease was reported for the first time in *A. aculeatus* TBRC 277 species. The CRISPR/Cpf1 system developed in this study highlights the feasibility of CRISPR/Cpf1 technology and could be envisioned to further increase the utility of the CRISPR/Cpf1 in facilitating strain improvements as well as functional genomics of filamentous fungi.

**Supplementary Information:**

The online version contains supplementary material available at 10.1186/s12896-021-00669-8.

## Background

Filamentous fungi are of great value in the commercial production of enzymes and heterologous proteins [1, 2], a wide range of primary and secondary metabolites such as organic acids and pharmaceuticals [3, 4], as well as bio-based chemicals, and biofuels [5, 6]. One of the ways to increase the fungi potential in commercial applications is by means of strain improvement via genetic engineering [7]. However, the conventional genetic engineering tools via classical mutagenesis and homologous recombination remain challenging due to additional complexity and time, as well as low efficiency, particularly in targeting multiple genes [8]. Therefore, the development of versatile genetic tools, e.g., CRISPR-based, for filamentous fungi modification would be of great value [9].

The rapid technological advances in low-cost high-throughput sequencing, as well as in CRISPR-Cas genome engineering technology, have accelerated research in several industrially and medically important *Aspergillus* species [10, 11]. Clustered regularly interspaced short palindromic repeats (CRISPR) and CRISPR-associated proteins (Cas), evolved as a defense system of bacteria to invading viruses. This system has become a versatile tool to solve the problem of low gene editing frequency in filamentous fungi [12]. CRISPR/Cas, particularly CRISPR/Cas9 from *Streptococcus pyogenes*, has become a tool in basic research, genetic improvement, and metabolic engineering [13]. In this system, a guide RNA (gRNA) or CRISPR RNA (crRNA) is used to direct Cas9 which targets sequence-specific DNA according to two simple rules: Watson-Crick base pairing between DNA target with the 5′-end guide sequence of crRNA, and the presence of a protospacer adjacent motif (PAM) 5′-NGG in the DNA target [14, 15]. Recently, applications of CRISPR/Cas9 in filamentous fungi have been shown in several fungal strains, e.g., *Aspergillus fumigatus* [16], *Aspergillus oryzae* [17], *Aspergillus niger* [7, 9, 18–20], *Aspergillus nidulans* [21, 22], *Trichoderma reesei* [23], *Neurospora crassa* [24], *Ganoderma lucidum* [25], and *Myceliophthora thermophila* [26]. In addition to Cas9 from *S. pyogenes*, Cpf1s derived from *Francisella tularensis* subsp. *novicida* U112 (FnCpf1), *Acidaminococcus* sp. BV3L6 (AsCpf1), and *Lachnospiraceae bacterium* (LbCpf1), are additional tools for genome editing. Cpf1 is distinct from Cas9 in terms of PAM sequence, structure of the guide RNA, and the DNA cleavage position [27].

CRISPR/Cpf1 is characterized as a novel class 2 (type V) system with distinct features compared to Cas9 [28]. It is a single RNA-guided endonuclease which recognizes a thymidine-rich protospacer-adjacent motif (PAM) and produces staggered cuts distal to the PAM site [29]. This type V CRISPR/Cpf1 system was first harnessed and shown to have robust genome editing activity in mammalian cell lines [27], as well as targeted mutations in plants and other eukaryotic cells [30]. Interestingly, Cpf1 is a dual nuclease that not only cleaves target DNA but also processes its own crRNA array [29, 31]. CRISPR/Cpf1-mediated DNA cleavage is guided by a short single crRNA (42–44 nt), in contrast to Cas9 that uses both crRNA and tracrRNA. More importantly, Cpf1 makes a staggered DNA double-stranded break resulting in a five-nucleotide 5′-overhang distal to the PAM site [29], whereas Cas9 creates blunt ends proximal to the PAM site [15]. However, both Cas9 and Cpf1 need a seed sequence at the PAM-proximal side of the protospacers, which is critical for DNA recognition and cleavage. With these advantages, Cpf1 system has been used for multiplex gene editing in mammalian cells, where up to four genes were simultaneously edited using just a single crRNA array spaced by direct repeats (DR) [29].

**Fig. 1:**
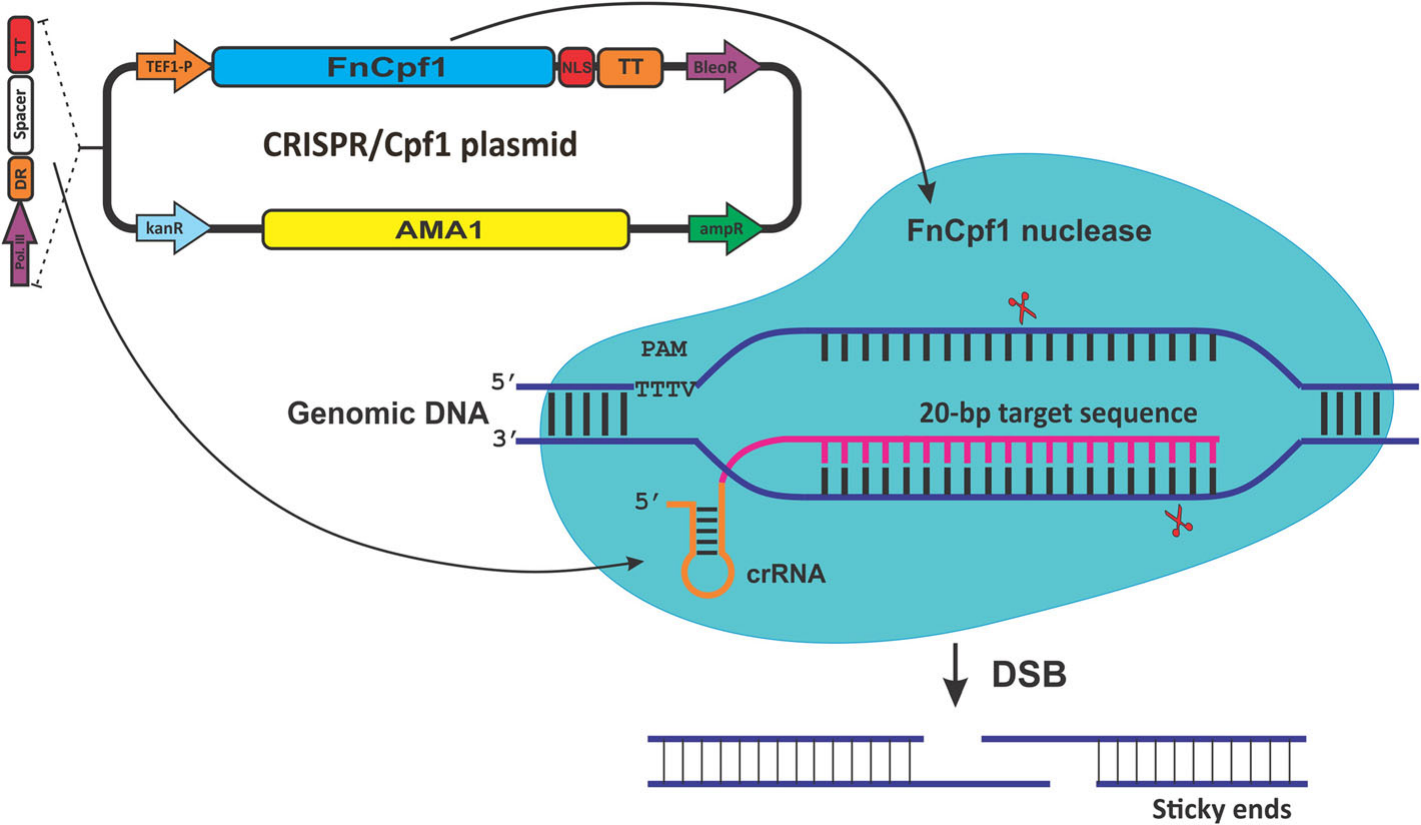
Schematic of CRISPR/Cpf1 in a single-plasmid system for use in fungal genome engineering application. In this study, FnCpf1 and crRNA are expressed under *A. nidulans* TEF1 Pol II promoter and *A. fumigatus* U3 Pol III promoter, respectively, to edit genome of the wild-type strain of *A. aculeatus* TBRC 277

Cpf1 nuclease and the crRNA are two essential components in the formation of an adjustable CRISPR toolbox for genetic manipulation (Fig. 1). In general, Cpf1 expression is driven by an RNA polymerase II promoter [32]. The guide RNAs are noncoding small RNAs with guide sequence at their 3′-end crRNA and are naturally expressed using an RNA polymerase III (Pol III) promoter [33]. However, due to the complexity and uncertainty of genome information, some endogenous Pol III promoters from filamentous fungi are difficult to identify or are not suitable for crRNA transcription [12]. Therefore, suitable crRNA expression cassettes are needed to construct efficient CRISPR-based tools. Until recently, several methods have been developed to enhance the expression of crRNAs by engineering different RNA processing machineries, including a self-cleavable ribozyme from a virus [34], the endogenous tRNA processing enzymes [35], and pre-crRNA array consisting of direct repeats [29]. These systems are used to generate crRNAs from a single primary polycistronic transcript driven by either Pol II or Pol III promoter. Among these methods, a single pre-crRNA array and a tRNA-based CRISPR system have been shown to boost multiplex genome-editing capability and efficiency without introducing exogenous ribonucleases or additional tracrRNA in the CRISPR/Cas9 system [21].

Over the past several years, various studies have demonstrated the great potential of CRISPR/Cpf1 as a tool for genome engineering, owing to user-designated site specificity of Cpf1 endonuclease activity and the simplicity of crRNA designs [27, 30]. Since the first harnessing of Cpf1 in human and animal cell lines [27], until recenty, AsCpf1 and LbCpf1 were reported to effectively catalyze oligonucleotide-mediated genomic site-directed mutagenesis and simultaneous gene deletions/insertions in a few filamentous fungi [36–38]. However, there have been no reports for the use of FnCpf1 from *F. tularensis* subsp. *novicida* U112 for genome editing in *Aspergillus* filamentous fungus despite its successful application in yeast [39–41]. Here, we established a single vector for the CRISPR/Cpf1 platform derived from FnCpf1 and a crRNA with the endogenous tRNA-processing system for CRISPR-mediated gene editing in *Aspergillus aculeatus* TBRC 277, a wild-type filamentous fungus. As a proof of principle, *pyrG* gene was used as the target gene for the CRISPR/Cpf1-mediated gene editing to generate uracil auxotrophic fungi.

## Results

### Construction of CRISPR/Cpf1 plasmid backbone

**Fig. 2:**
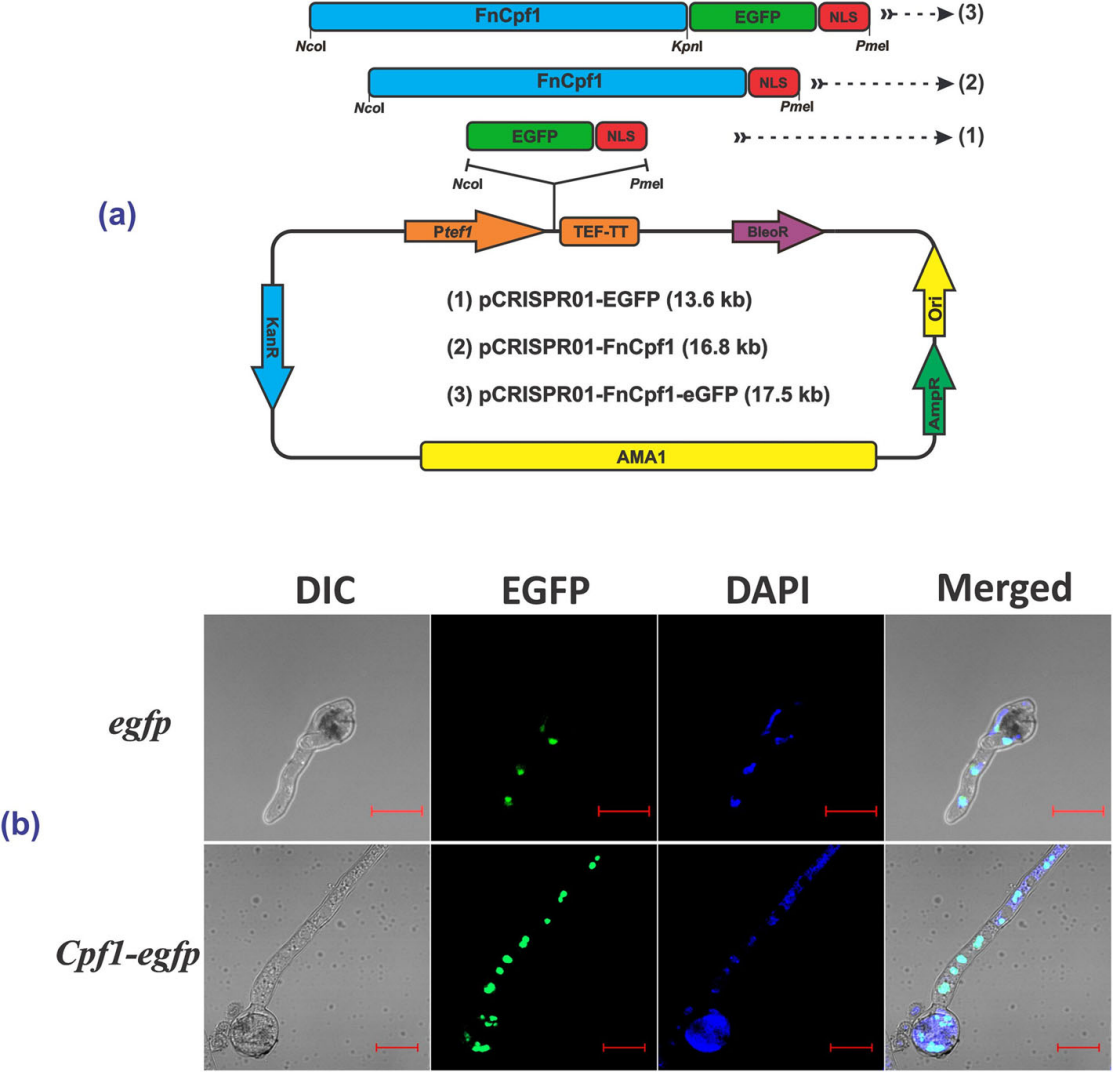
Schematic of vector constructions for heterologous FnCpf1 expression and confocal microscopy assessment of the subcellular localization of the recombinant eGFP and Cpf1-EGFP fusion proteins in *A. aculeatus* TBRC 277. **a** The constructed vectors of (1) pCRISPR01-EGFP, expression of enhanced green fluorescence protein (EGFP), to test the tef1 promoter activity, (2) pCRISPR01-FnCpf1, for subsequent crRNA cassette insertion carrying a protospacer pyrG-targeted locus, (3) pCRISPR01-FnCpf1-EGFP, expression of Cpf1-EGFP, to detect Cpf1 fusion protein. **b** Subcelullar localization of FnCpf1 as EGFP-fused protein in *A. aculeatus* TBRC 277. Nuclei were visualized by DAPI staining (blue). Scale bar = 10 μm. DAPI, 4′,6-diamidino-2-phenylindole; EGFP, enhanced green fluorescent protein

To establish the CRISPR/Cpf1 system in *A. aculeatus* TBRC 277 (Fig. 2a), an expression plasmid carrying the P_TEF1_-Cpf1-_TEF1_TT cassette and bleomycin selection marker was initially constructed. For Cpf1 gene, we codon-optimized the *F. tularensis* subsp. *novicida* Cpf1, so called *FnCpf1*, and attached a SV40 nuclear localization signal (NLS) fragment at the C-terminal to ensure successful nuclear compartmentalization in the fungal host. Specifically, construction of CRISPR/Cpf1 backbone carrying 5.5 kb autonomously replicating plasmids (AMA1) was successfully obtained by removing Cas9 from pFC333 plasmid [22], followed by MCS insertion that allows for expression of a 3.93 kb Cpf1-SV40 NLS gene fusion controlled by a constitutive *A. nidulans* TEF1 promoter. The successfully constructed plasmid, referred to as pCRISPR0 (Supplementary Fig. S1), was verified by Sanger sequencing. Then, insertion of kanamycin resistant gene cassette at the *Bgl*II-site was performed. This was confirmed by positive selection on LB agar containing 50 μg/ml kanamycin, as well as 25 μg/ml ampicillin; and also by *Hin*dIII digestion of recombinant plasmids. The obtained plasmid was named pCRISPR01 (Supplementary Fig. S1**)** and used throughout this study as the vector backbone for FnCpf1 and crRNA expressions.

### Expression of codon-optimized eGFP and eGFP-Cpf1 in *A. aculeatus* TBRC 277

To test the expression and localization of the recombinant FnCpf1, the enhanced green fluorescent protein (eGFP) and FnCpf1-eGFP fusion, with attached SV40 NLS, were used as the gene reporter. These were constructed by ligation of each DNA fragment to the *Nco*I/*Pme*I-site of pCRISPR01, yielding pCRISPR01-eGFP and pCRISPR01-FnCpf1-eGFP plasmids, respectively. The positive clones of each construct were confirmed by restriction analyses and further verified by sequencing. The obtained vectors, pCRISPR01-eGFP and pCRISPR01-FnCpf1-eGFP, were individually transformed into *A. aculeatus* TBRC 277 host. Positive transformants were further confirmed by colony PCR, followed by single spore isolation hereafter referred to as *egfp* and *Cpf1*-*egfp* accordingly.

The expression of eGFP and Cpf1-eGFP was detected after culturing TBRC 277 transformants in PDB for 24 h. Western blot detected specific protein bands of approximately 28 kDa and 180 kDa as predicted for eGFP and Cpf1-eGFP, respectively (Supplementary Fig. S2), suggesting that codon-optimized FnCpf1 was expressed in *A. aculeatus* TBRC 277. Additionally, to investigate whether the SV40 nuclear localization signal targeted FnCpf1 to the nucleus, the transformants were further assessed using confocal microscopy. The result showed that both transformants, *egfp* and *Cpf1-egfp*, showed green fluorescence signal when compared to the WT, after overnight incubation in PDB medium with bleomycin. Furthermore, these recombinant fungi were also stained with 4,6-diamidino-2-phenylindole (DAPI) and showed that the majority of the eGFP signals were overlapped with nuclei, suggesting that FnCpf1 correctly localized to the nucleus of *A. aculeatus* TBRC 277 (Fig. 2b). Finally, to obtain the expression vector for further genome engineering in TBRC 277, we also cloned just FnCpf1-NLS without eGFP to generate pCRISPR01-FnCpf1 plasmid.

### Construction of guide RNA (crRNA)

CRISPR/Cpf1 system requires crRNA consisting of a 20-bp direct repeat (DR) followed by a 20–23 bp protospacer to direct Cpf1 to cleave sequence-specific target [41]. In this study, *pyrG*, an essential gene for the biosynthesis of uridine, was chosen as the target gene. As *pyrG* mutants undergo auxotrophic selection, its inactivation can be observed by growing transformants in minimal medium containing 5-fluoroorotic acid (5-FOA) [42, 43]. PyrG converts 5-FOA into fluoroorotidine monophosphate which is subsequently converted into fluorodeoxyuridine by ribonuclease reductase. Fluorodeoxyuridine is a pyrimidine analog that is rapidly converted to 5-fluorouracil, a suicide inhibitor of the thymidylate synthase, and therefore inhibits nucleic acid synthesis, leading to cell death; however, 5-FOA is non-lethal in the absence of *pyrG* [9, 44].

**Fig. 3:**
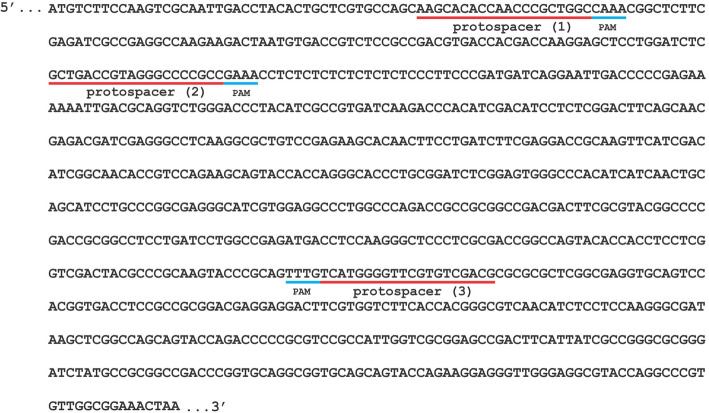
The complete sequence of pyrG gene of *A. aculeatus* TBRC 277 (GenBank accession number MN364695). The locations of the three selected protospacers are indicated by the red lines, with the corresponding 5′-TTTN-3′ PAM in blue (putative intron 158–238; 81 bp)

To perform this experiment, first, we needed to validate the sequence of *A. aculateus* TBRC 277 *pyrG* gene from the genomic draft sequence as generated by Next Generation Sequencing (NGS). The *pyrG* gene was successfully PCR amplified and sequenced (Fig. 3). The 915-bp amplified sequence was identified to encode orotidine 5′-phosphate decarboxylase (*pyrG*); it showed 93.63% sequence identity to *A. aculeatus* ATCC 16872 and had a 81-bp putative intron at the positions 158–238. Secondly, since FnCpf1 could be programmed to edit multiple target sites [29], this aspect was tested by selecting three out of eight predicted protospacers with the following criteria: no off-targets and highest specificity score (100%). Three protospacer guide sequences were obtained targeting positions 59-bp (pyrG-1), 167-bp (pyrG-2), and 648-bp (pyrG-3) downstream of the *pyrG* start codon.

Next, due to the lack of well-identified RNA Pol III promoters in *A. aculeatus*, we instead employed a U3 Pol III promoter derived from *A. fumigatus* to generate a single crRNA construct, i.e. a 182-bp crRNA-flanking tRNA fusion transcript (GenBank KT031983.1) [21]. Using this approach, the endogenous tRNA processing system was expected to cleave both ends of the tRNA precursor and release a single crRNA with the aid of RNAses Z and P [35]. In this study, U3 Pol III promoter and its terminator, together with tRNA-sgRNA(Cas9)-tRNA motif originating from pFC902, were successfully amplified and inserted into pJET1.2B resulting in pJET-U3P sgRNA (Table 1). This plasmid served as a template for the subsequent PCR reactions to generate six DNA fragments, in which each of the associated fragments were assembled with *Bgl*II-cut pOK12 by Gibson Assembly (see Methods)*.* The obtained tRNA-crRNA-tRNA Cpf1-associated cassettes in pOK12 specifically referred to as crRNA-tRNA-pyrG-1, crRNA-tRNA-pyrG-2, and crRNA-tRNA-pyrG-3 which corresponded to the sequence-specific protospacers pyrG-1, pyrG-2, and pyrG-3, respectively. To finalize the CRISPR/Cpf1 construction, each of the purified crRNA-pyrG cassettes successfully incorporated the *Bgl*II-cut pCRISPR01-FnCpf1 to obtain the plasmid constructs named pCRISPR01-FnCpf1-pyrG-1, pCRISPR01-FnCpf1-pyrG-2, and pCRISPR01-FnCpf1-pyrG-3. All of these plasmids were verified by sequencing and by *Bgl*II digestions, which generated 16.8-kb and 0.9-kb DNA fragments representing the vector pCRISPR01-FnCpf1 and crRNA-pyrG inserts, respectively **(**Supplementary Fig. S3**)**.

**Table 1:** Plasmids and fungal strain used in this study

Plasmids/Strain	Genotype	Reference
**Plasmid**		
pFC902	Amp^R^, *pyrG,* AMA1, U3-AF crRNA Cas9	[21]
pJET1.2B-Km^R^	Amp^R^, kan^R^	This study
pJET-U3P sgRNA	Amp^R^, U3-AF sgRNA Cas9	This study
pFC333	Amp^R^, ble^R^, AMA1, TEF-Cas9	[22]
pCRISPR0	Amp^R^, ble^R^, AMA1	This study
pCRISPR01	Amp^R^, ble^R^, kan^R^, AMA1	This study
pCRISPR01-eGFP	Amp^R^, ble^R^, kan^R^, AMA1, TEF1-EGFP	This study
pCRISPR01-FnCpf1	Amp^R^, ble^R^, kan^R^, AMA1, TEF1-FnCpf1	This study
pCRISPR01-FnCpf1-eGFP	Amp^R^, ble^R^, kan^R^, AMA1, TEF1-Cpf1-EGFP fusion	This study
pOK-U3P crRNA pyrG-1	Kan^R^, U3-AF crRNA-pyrG-1	This study
pOK-U3P crRNA pyrG-2	Kan^R^, U3-AF crRNA-pyrG-2	This study
pOK-U3P crRNA pyrG-3	Kan^R^, U3-AF crRNA-pyrG-3	This study
pCRISPR01-FnCpf1-pyrG-1	Amp^R^, ble^R^, kan^R^, AMA1, TEF-Cpf1, crRNA-pyrG-1	This study
pCRISPR01-FnCpf1-pyrG-2	Amp^R^, ble^R^, kan^R^, AMA1, TEF-Cpf1, crRNA-pyrG-2	This study
pCRISPR01-FnCpf1-pyrG-3	Amp^R^, ble^R^, kan^R^, AMA1, TEF-Cpf1, crRNA-pyrG-3	This study
**Strain**		
*A. aculeatus* TBRC 277	Wild type	[1]

### CRISPR-crRNA complex induced *pyrG*-targeted gene disruption in *A. aculeatus* TBRC 277

**Fig. 4:**
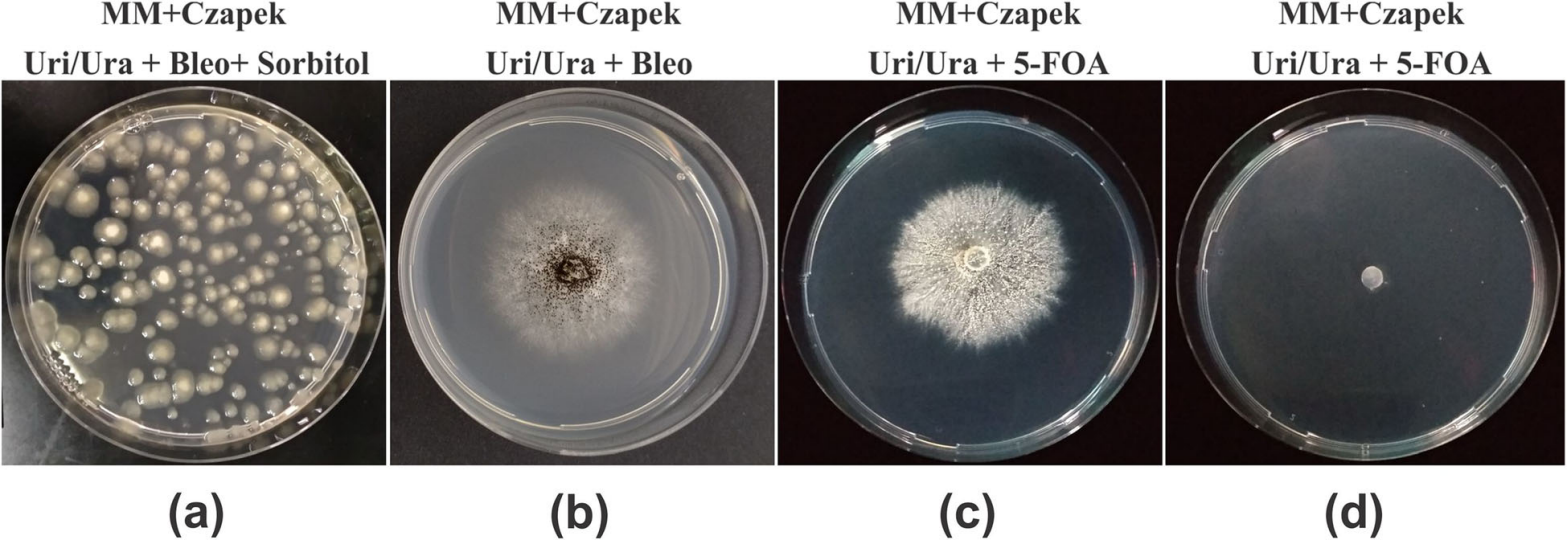
An example of *A. aculeatus* TBRC 277 protoplasts transformed with CRISPR/Cpf1 plasmids. **a** pCRISPR01-FnCpf1-pyrGs (with crRNA specific targeting pyrG gene) on primary selective transformation medium containing 1.2 M sorbitol + 10 mM uri/ura + 50 μg/ml bleomycin, **b** a single colony selected from panel a, was then cultured on new minimal medium containing 10 mM uri/ura+ 50 μg/ml bleomycin, (**c**) secondary selection from panel b, re-cultured on minimal medium containing 10 mM uri/ura and 1.5 mg/ml 5-FOA. **d** pCRISPR01-FnCpf1 encoding only Cpf1 (without crRNA targeting pyrG), after growing on secondary selective medium containing 5-FOA, no colony was observed (control). Uri: uridine, Ura: uracil

This study relies on the ability of Cpf1/crRNA-complex targeting *pyrG* locus to induce DSB, followed by NHEJ-repair mechanism. While some methods employ either Cas9 or Cpf1 expression in vivo but crRNA preparation in vitro which requires laborious steps for crRNA transcript preparation as well as an additional transformation step when introducing the crRNA. This leads to low transformation efficiencies due to crRNA stability issues [9, 14]. We therefore opted for in vivo preparation both of crRNA and Cpf1 in a single plasmid (see Methods). To test whether heterologous expression of the CRISPR/Cpf1 system is able to induce *pyrG*-targeted DSB in *A. aculeatus* TBRC 277 chromosome, as well as to explore the general utility of RNA-guided genome editing in fungal cells, three pCRISPR01-FnCpf1-pyrGs plasmids expressing Cpf1 and three different crRNA guides were independently transformed into *A. aculeatus* TBRC 277 protoplasts. Next, *pyrG-*mutant selection was performed by a two-step selection by first randomly selecting bleomycin-resistant colonies **(**Fig. 4a**)** and re-culture the colonies on new MM+Czapek-Dox plates with bleomycin and Uri/Ura supplementation, referred as the primary selection plate, but in the absence of sorbitol (Fig. 4b). Then, the colonies were grown on the secondary MM+Czapek-Dox plates supplemented with Uri/Ura as well as 5-FOA (Fig. 4c**)**. The colonies that survived on this medium were considered mutant auxotroph [45]. For a negative control, the protoplast was transformed with empty vector or only Cpf1-bearing plasmid, and no colony was observed on the secondary selection plates (Fig. 4d). Our preliminary study showed that selective medium for protoplast-mediated transformation comprised of a mixture of sorbitol and 5-FOA gave rise to false-positives. Therefore, a two-step selection was required to remove fungal clones without the CRISPR plasmid. Using this approach, several bleomycin-resistant colonies obtained from the primary selection were not viable on secondary selective medium containing 5-FOA considered as non-mutated *pyrG* transformants.

Additionally, we also tested the transformation efficiency of Cpf1-bearing plasmids (in pCRISPR01-FnCpf1) which was 30 colonies/μg of DNA (Supplementary Fig. S4). This was slightly lower than that of the empty vector without Cpf1 (37.5 colonies/μg of plasmid DNA). This may be due to size of the plasmid since the Cpf1-bearing plasmid is approximately 4–5 kb larger. However, the effect of the constitutive Cpf1 expression from the AMA1-based plasmid may also contribute to the minor difference in transformation efficiency. Similar results were also reported in *A. nidulans* [36], and *Ashbya gossypii* [37] expressing LbCpf1. However, the potential toxicity effect of FnCpf1 was reported in yeast expressing multicopy plasmid [40].

**Fig. 5:**
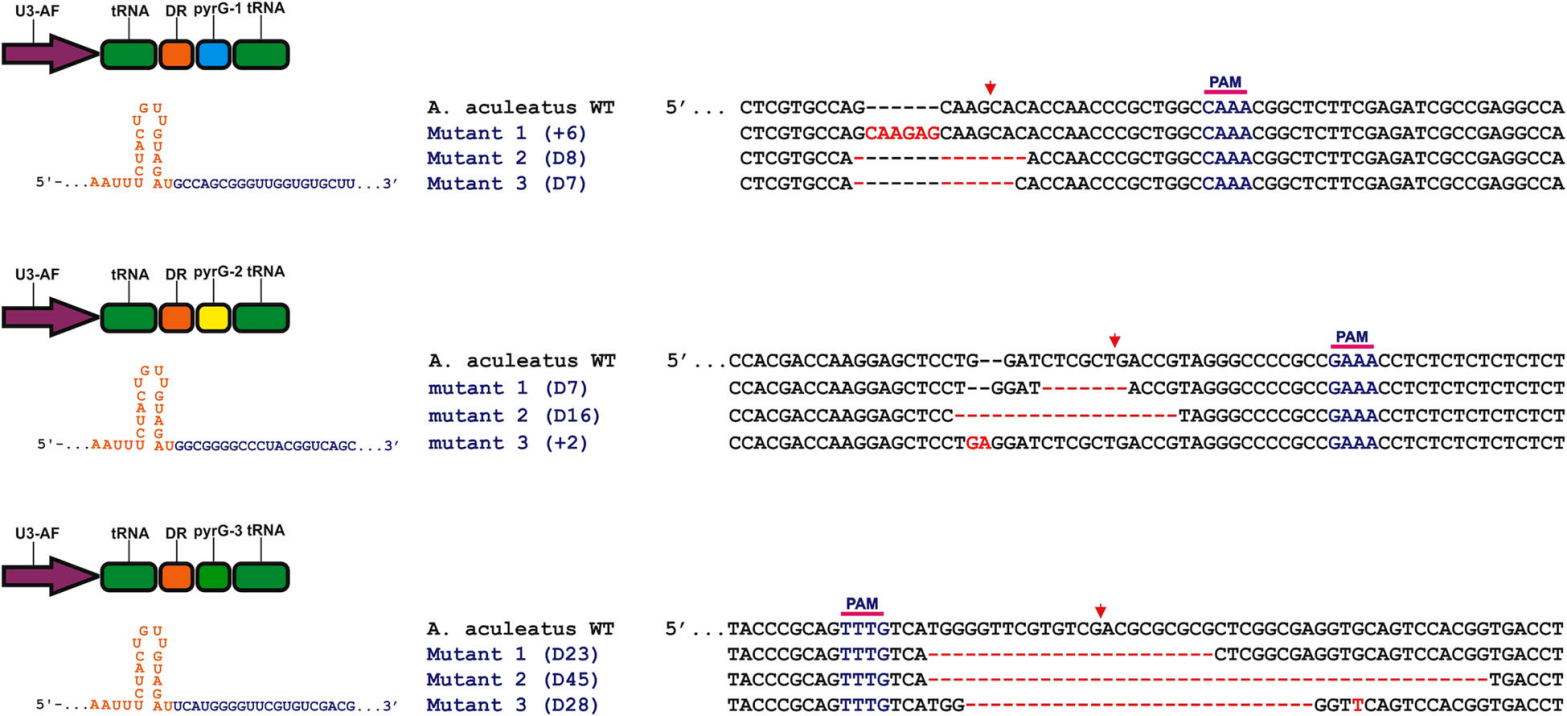
Indels of pyrG gene in *A. aculeatus* TBRC 277 genome transformed with CRISPR/Cpf1 plasmids with three different crRNAs. Red arrow indicates putative cleavage sites; red dashes, deleted bases; red bases, insertions or mutations

To confirm whether the obtained colonies were auxotrophic mutants, PCR was performed to detect the presence of introduced exogenous CRISPR plasmid followed by sequencing of the pyrG gene. Using this approach, each randomly selected clones exhibited the presence of indels with 100% efficiency in every pyrG locus (Supplementary Table S1). Sequencing of the *pyrG*-specific locus revealed that three out of ten randomly selected mutant of each auxotroph contained *pyrG* indels (Fig. 5). These results indicated that Cpf1-crRNA complex induces DSB and endogenous error-prone repair that leads to indels of the *pyrG* gene in *A. aculeatus* TBRC 277*.*

Moreover, these DNA sequences were further analyzed. The results showed that mutants carrying either crRNA-pyrG-1 or crRNA-pyrG-2, typically had small insertions or deletions in the range of 2–6 bp and 7–16 bp, respectively. Interestingly, in a mutant carrying crRNA-pyrG-1, we noticed a 6-bp insertion in the exon of the pyrG-targeting cleavage site; this small in-frame insertion somehow could abolish PyrG activity. Additionally, for mutants carrying crRNA-pyrG-3, their deletions were in the range of 23–28 bp. These similar observations were also reported in other fungi [17, 23], plant [46], and animal cell line [27]. However, large DNA insertions with sizes greater than 100-bp were also found in which polyketide synthase gene (*pksP*) was targeted in *A. fumigatus* [16].

## Discussion

CRISPR-Cas based systems have been broadly applied for use in targeted genome modification of cells or organisms due to their programmable sequence specificity. More recently, the CRISPR-Cas based system is no longer limited to Cas9 from *S. pyogenes*. As an alternative to Cas9, two Cas nuclease genes originating from *Acidominococcus* and *Lachnospiraceae* that belong to the Cpf1 family have been reported [27]. The introduction of CRISPR/Cpf1 system provides another option to the CRISPR toolbox [47]. So far, applications of CRISPR-Cas in filamentous fungi have focused mostly on Cas9 [28, 32, 48]. Here, we presented data of another type of Cpf1, called FnCpf1, and demonstrated the NHEJ pathway involvement in the repair of Cpf1-induced DNA DSBs in a non-model filamentous fungus, *A. aculeatus* TBRC 277.

The success of genome editing by CRISPR/Cpf1 system requires the heterologous expression of the codon-optimized Cpf1 gene fused with a NLS (PKKKRKV) to efficiently target the fused protein into the cell nucleus. To date, studies of the CRISPR/Cas9 system have been applied for genome engineering applications in various filamentous fungi such as *A. fumigatus* [16], *A. oryzae* [17], *A. niger* [7, 9, 18–20], *A. nidulans* [21, 22], *T. reesei* [23], *N. crassa* [24], *G. lucidum* [25], and *M. thermophila* [26]. These studies were performed by taking advantage of the Cas9 in vivo expression strategy using various types of promoters such as *tef1, gpdA, trpC, pdc,* and *cbh1*. Until recently, for CRISPR/Cpf1 system, only LbCpf1 from *L. bacterium* and AsCpf1 from *Acidaminococcus sp.* were reported to effectively catalyze oligonucleotide-mediated genomic site-directed mutagenesis in the filamentous fungi *A. nidulans* and *M. thermophila*, respectively [36, 38].

crRNA together with FnCpf1 were expressed via a single vector in the host cell, hence, it is not necessary for in vitro crRNA preparation which is laborious and time consuming [12]. In this study, to increase the ability of *A. fumigatus* U3 Pol III promoter to drive the functional transcription of crRNA, we chose a tRNA-processing system to yield a mature crRNA transcript rather than introducing any additional RNases along with Cpf1/crRNA components. This strategy was also employed for CRISPR/Cpf1 based multiplex genome editing applications in another filamentous fungi [21]. Here, we successfully produced the monocistronic tRNA-processing system and verified its ability to edit *pyrG* gene in the host TBRC 277 genome. Hence, this report demonstrated the successful in vivo expression and assembly of FnCpf1 and crRNA to form functional FnCpf1-crRNA ribonucleoprotein (RNP) complexes. These complexes serve as RNA-guided nucleases to specifically target and cleave genomic DNA. In addition, these results also suggested that a 20-bp protospacer sequence is sufficient in CRISPR/Cpf1 based system, in contrast to the previous reports using a 23-bp protospacer [29, 36]. Our study therefore supports the previous report that the Cpf1 in complex with the crRNA and double-stranded DNA target was dictated by only the first 20-bp of heteroduplex formation of crRNA and target DNA [49].

We first developed CRISPR/Cpf1 vector specifically for FnCpf1 nuclease, as a proof of concept for gene editing in *A. aculeatus* species. To our knowledge, the use of FnCpf1 nuclease gene for targeted gene editing has not been reported in any Aspergillus species despite its successful applications in bacteria [50], yeast [40], plant [30], and animal cell lines [29]. In this study, we have shown that the CRISPR/Cpf1 system is able to site-specific edit *A. aculeatus* chromosome at three different loci within the *pyrG* gene by constructing specific protospacer guides. However, for genes that are located in a region of low transcriptional activity this would probably reduce the editing efficiency and require optimization [23]. Although it is advisable to carry out off-target mutation analyses by PCR and sequencing, local BLAST analyses of each guide showed no off-targets. Interestingly, indels obtained from all *pyrG* mutants retain the PAM site since FnCpf1 cleaves target DNA at a distal position, and this report is in accordance with the previous studies in plants [46, 51]. In contrast, Cas9-mediated NHEJ usually destroys the PAM site due to its proximity to the cleavage site thus preventing the possibility for future edits. Since we employed AMA1-based CRISPR/Cpf1 expression vector for transient expression of both FnCpf1 and crRNAs, this would allow us to perform iterative genome editing. This iterative process would allow for targeted gene disruption of multiple targets as well as marker-free genome editing, thus greatly improving the engineering throughput. Some Cpf1 features described above make Cpf1 has several advantageous over Cas9 for gene editing applications even though in some cases both Cpf1 and Cas9 achieved comparable targeting efficiencies [41]. In addition, we envision that gene editing by CRISPR/Cpf1 could be efficiently used as a powerful tool and would enhance the range of possible target sites because of its unique PAM sequence, as well as simplicity in designing crRNA cassettes, when compared to the well-studied CRISPR/Cas9 system.

## Conclusions

In summary, we have demonstrated the use of CRISPR/Cpf1 mediated gene editing technology in the wild type strain of *A. aculeatus* TBRC 277. Using this system, the *pyrG* gene involved in uridine biosynthesis pathway was successfully edited to generate an auxotrophic strain. The use of FnCpf1 as an alternative class II (type V) nuclease was reported for the first time in *A. aculeatus* species. The CRISPR/Cpf1 system developed in this study highlights the feasibility of CRISPR/Cpf1 technology and could be envisioned to further increase the utility of the CRISPR/Cpf1 in facilitating strain improvements as well as functional genomics of filamentous fungi.

## Materials and methods

### Strains, media, and culture conditions

*Escherichia coli* strain DH5α (Novagen, USA) served as cloning host and for propagating all plasmids. *E. coli* bearing plasmids were grown in liquid or with 1.5% agar in Luria-Bertani (LB) medium plus ampicillin (100 μg/ml) or kanamycin (50 μg/ml) when required. Recombinant plasmids were purified from *E. coli* according to QIAquick spin miniprep kit (QIAGEN, Germany). Wild type (WT) strain of *A. aculeatus* TBRC 277 (= BCC 199), obtained from the Thailand Bioresource Research Center, was used as the host for CRISPR/Cpf1 expression and gene editing experiments. The *pyrG* gene, encoding orotidine-5′-monophosphate decarboxylase, was retrieved from a complete genome sequence of *A. aculeatus* TBRC 277. The fungus was grown at 30 °C for 2–4 days on potato dextrose agar (PDA) medium for sporulation or potato dextrose broth (PDB) for mycelia propagation. Minimal medium (MM) plus Czapek-Dox (3% sucrose, 0.3% NaNO_3_, 0.1% KH_2_PO_4_, 0.05% MgSO_4_.7H_2_O, 0.05% KCl, 0.001% FeSO_4_.7H_2_O) containing 1.2 M sorbitol and 50 μg/ml of bleomycin was used as selective medium for protoplast-mediated transformation [52]. MM plus Czapek-Dox medium was supplemented with 10 mM uridine (Uri), 10 mM uracil (Ura), and 1.5 mg/ml 5-fluoroorotic acid (5-FOA) (Sigma Aldrich, USA) when required. Plasmids pFC333 and pFC902 [21, 22] were used as templates for constructing CRISPR/Cpf1 and crRNA based plasmids, respectively. Plasmids pJET1.2/Blunt (Thermo Fisher, USA) and pOK12 [53] served as cloning vectors.

### Plasmid constructions

All oligonucleotide primers used in this study are shown in Supplementary Table S2. All PCR reactions for cloning purposes were performed for 35 cycles with Phusion high-fidelity DNA polymerase (Thermo Fisher, USA) using touch-down PCR with annealing temperature starting from 65 °C, with a decrement of 1 °C per cycle for the first 10 cycles followed by a constant annealing temperature at 55 °C up to 35 cycles. Standard reaction volumes were 50 μl including 1x Phusion HF or GC Buffer, 0.2 mM dNTPs, 0.5 μM primers (IDT, USA), 1 unit of Phusion DNA Polymerase, 10–50 ng of genomic DNA or plasmid as the template. All PCR products were purified using Qiaquick gel extraction and PCR clean-up kits (Qiagen, Germany) according to manufacturer’s instruction. All expression vectors were assembled by standard molecular cloning or Gibson assembly methods [54].

All plasmids used in this study are listed in Table 1. A detailed schematic map of CRISPR/Cpf1 based expression vector construction is illustrated in Supplementary Fig. S1. pFC333 was used to establish CRISPR/Cpf1-based expression vectors [22]. Briefly, to create multiple cloning sites (MCS), a 421-bp of TEF1 terminator from pFC333 was amplified by using a pair of primers pFC333-TT-NcoI-F and pFC333-TT-BamHI-R. The amplified DNA fragment was digested by *Nco*I/*Bam*HI and ligated to a 11.1-kb of *Nco*I/*Bam*HI-cut pFC333 plasmid to generate expression vector backbone named pCRISPR0. Additionally, a 1.4-kb *Bam*HI/*Bgl*II-cut kanamycin resistance gene cassette, amplified from pJET1.2B-Km by using KanR-Bgl2-F and KanR-R primer pairs, was ligated to *Bgl*II-cut pCRISPR0, generating pCRISPR01 vector (Supplementary Table S3). The presence of the intended MCS sequences flanked by *Aspergillus nidulans* TEF-1 promoter and terminator in the pCRISPR01 was verified by sequencing.

The sequence of *F. tularensis* subsp. *novicida* U112 *Cpf1* gene, *FnCpf1*, was obtained from pY001 plasmid [27]. A 3.9-kb of *FnCpf1* gene (Supplementary Table S3) with its C-terminal fused to SV40 NLS was optimized for translation on the basis of codon frequency in *A. aculeatus* (http://www.kazusa.or.jp/codon/cgi-bin/showcodon.cgi?species=5053&aa=1& style=N) and synthesized (GenScript, USA) to obtain pUC57-FnCpf1 plasmid. Then, the FnCpf1-SV40 fusion gene was PCR-amplified from the plasmid using a pair of primers FnCpf1-NcoI-F and FnCpf1-PmeI-R, followed by *Nco*I/*Pme*I digestion and ligation to pCRISPR01 to obtain a plasmid named pCRISPR01-FnCpf1 (Table 1, Fig. 2a).

To detect the expression and localization of Cpf1, enhanced GFP (eGFP) gene was fused in-frame to Cpf1 at the C-terminal together with SV40 NLS at the 3′ end of the fused sequence to generate plasmid pCRISPR01-FnCpf1-eGFP (Supplementary Table S3, Fig. 2a). This was carried out by amplification of pUC57-FnCpf1 and pUC57-EGFP with a pair of primers FnCpf1-NcoI-F/FnCpf1-Kpn-R and EGFP-NK-F/ EGFP-SV40-Pme-R, respectively, followed by restriction enzyme digestion (*Nco*I/*Kpn*I for FnCpf1 and *Kpn*I/*Pme*I for eGFP) and ligation. In addition to Cpf1 and Cpf1-EGFP fusion constructs, the expression vector bearing only eGFP gene was also employed as a control by amplification of eGFP in pUC57-EGFP using the same primers used for eGFP fusion construction, but the amplified PCR product was digested by *Nco*I/*Pme*I prior to ligation with plasmid pCRISPR01.

### Protoplast-mediated transformation

All CRISPR/Cpf1 vectors were transformed into the recipient *A. aculeatus* TBRC 277 using the protoplast-mediated transformation method [55, 56]. Briefly, spore suspension (10^5^ spores/ml) was inoculated into 50-ml of PDB and incubated at 30 °C with shaking at 250 rpm for 10–12 h. Mycelia were collected by filtration and washed with sterilized distilled water, then suspended in 5-ml protoplast buffer (1.2 M sorbitol, 100 mM potassium phosphate buffer, pH 5.6) together with enzyme mixtures (2 mg/ml β-glucoronidase, 10 mg/ml driselase, 20 mg/ml lysing enzyme/Glucanex). The mixture was incubated at 30 °C with gentle shaking at 100 rpm for 4–5 h. Protoplasts were separated from undigested mycelia by using Falcon Cell Strainer with 40 μm porosity (Thermo Fisher, USA), then gently rinsed with Wash Buffer (1.2 M sorbitol, 10 mM Tris HCl pH 7.5) followed by centrifugation at 3000 x *g* for 5 min. The protoplast pellets were resuspended in STC buffer (1.2 M sorbitol, 10 mM Tris-HCl pH 7.5, 50 mM CaCl_2_) to achieve a concentration of 10^7^–10^8^ protoplasts/ml.

Protoplast-mediated transformation (PMT) was carried out as described previously [9]. Specifically, 10-μg of purified DNA in 20 μl (500 ng/μl) was mixed with 100 μl of protoplast suspension (10^7^-10^8^ protoplast/ml) and incubated on ice for 50 min. Subsequently, 1.25 ml of PCT buffer (25% PEG 6000, 50 mM CaCl_2_, 10 mM Tris HCl pH 7.5) was added to the mixture followed by incubation at room temperature for additional 20 min. Next, 1.25 ml of STC buffer was added, followed by addition of 5 ml PDB in 1.2 M sorbitol. The solution was centrifuged at 3000 x *g* for 10 min and the supernatant was discarded. Next, 2 ml of STC buffer was added to the cell pellet, and gently mixed with 30 ml of preheat MM+Czapek-Dox soft agar medium at 45 °C. Approximately 5-ml of mixture was overlayed on the high osmotic pressure MM+Czapek-Dox plates with 1.2 M sorbitol containing 50 μg/ml bleomycin (InvivoGen, USA) and Uri/Ura, followed by incubation at 30 °C for 3-5 days. Fungal transformants were selected and purified to obtain single colonies onto new MM+Czapek-Dox containing 50 μg/ml bleomycin and Uri/Ura. For *pyrG* mutant selections, fungal transformants were selected on MM+Czapek-Dox plates containing 5-FOA and Uri/Ura.

### Fluorescence microscopy imaging and analysis

For recombinant fungi bearing pCRISPR01-FnCpf1-eGFP, bleomycin-resistant colonies were selected and purified to obtain single colonies followed by fluorescent microscopic assessment [57, 58]. Specifically, conidia were inoculated into 750 μl PDB medium containing 25 μg/ml antibiotics bleomycin and grown statically for 12–14 h at 30 °C. Before imaging, hyphae were washed with 1x PBS pH 7.4 and incubated with 50 μg/ml 4,6-diamidino-2-phenylindole (DAPI) (Sigma Aldrich, USA) for 30 min. The image was captured by Confocal Laser Scanning Microscope (LSM) 800 with Airyscan 2 (Carl Zeiss, Germany) with a 63x oil immersion objective lens. Image handling and quality optimization were performed using ZEN 2.1 imaging software (Carl Zeiss, Germany).

### Western blotting

Expression of FnCpf1 in *A. aculeatus* TBRC 277 was also detected by western blot as described [16, 59]. Specifically, total proteins from recombinant *A. aculeatus* mycelia was extracted by first inoculating 10^5^ conidia in 50-ml PDB medium with bleomycin (100 μg/ml) and incubated for 24 h at 30 °C with 250 rpm on a rotary shaker. Then, the mycelia were collected by filtration with Whatman Grade 41 filter paper (Sigma Aldrich, USA) followed by gently blotting with tissue paper to remove the remaining liquid media. The mycelia were ground into fine powders under liquid nitrogen with a mortar and pestle. Subsequently, approximately 200 mg of mycelia powders was added into an eppendorf tube containing 500 μl ice-cold extraction buffer (50 mM HEPES pH 7.4, 137 mM NaCl, 10% (v/v) glycerol 99.5%) with freshly added cOmplete mini protease inhibitor cocktail (Sigma-Aldrich, USA), followed by gently mixing for 5 min. The slurry was incubated on ice for additional 10 min, and the supernatant which contains a total protein was collected in a new tube after centrifugation at 14,000 x g for 15 min at 4 °C. The protein quantity was determined by Bradford method [60] using protein assay dye reagent (Bio-Rad, USA).

Approximately 20 μg of crude protein extracts was loaded in each lane for a 10% SDS-PAGE [16, 59]. After electrophoresis, proteins were transferred to a 5.5 × 8.5 cm nitrocellulose membrane with 0.2 μm pore size (Bio-Rad, USA) by using a Mini Trans-Blot Immersion (Bio-Rad) in 38.90 mM glycine, 47.88 mM Tris base, 0.0375% (w/v) SDS and 20% methanol at 90 V for 70 min. Then the membrane was soaked in phosphate buffer saline (PBS) for 5 min, then incubated in blocking buffer (PBS with 0.5% Tween 20 (PBST), 4% (w/v) non-fat dry milk) for 2 h at room temperature on shaking incubator with gentle agitation. Subsequently, the membrane was probed with anti-GFP mouse monoclonal antibody (Thermo Fisher, USA) or FnCpf1 mouse monoclonal antibody (Genscript, USA) in PBST buffer at 1:1000 dilution for 1 h. The membrane was washed with PBST buffer twice for 5 min each, then added with a 1:1000 dilution of anti-mouse IgG conjugated with alkaline phosphatase (Thermo Fisher, USA) in PBST buffer. The membrane was then washed twice with PBST buffer for 5 min each and developed using a 1-Step NBT/BCIP chromogenic substrate (Thermo Fisher, USA) by incubating the membrane for 5–15 min or until the desired color developed.

### Development of a single crRNA targeting *pyrG* gene in *A. aculeatus* TBRC 277

#### *pyrG* coding sequence validation

*A. aculeatus* TBRC 277 genomic DNA was extracted using Wizard Genomic DNA Purification Kit (Promega, USA). The *pyrG* gene from *A. aculeatus* TBRC 277 genomic DNA was PCR-amplified using pyrG-F and pyrG-R primers (Supplementary Table S2). The amplified fragment was purified and cloned into pJET1.2/B vector (Thermo Fisher, USA). Positive clones carrying DNA inserts were sequenced from both strands. Nucleotide sequence of *pyrG* obtained were pairwise-aligned to the sequence previously obtained by next-generation sequencing (NGS). The consensus sequence was submitted to GenBank under accession number MN364695 and used as template in selecting protospacer guide sequence (Table 2).

**Table 2:** Protospacer sequences selected for the gene editing of *A. aculeatus* TBRC 277

Spacer ID	Gene target	Genomic target sequence (5′> 3′)	PAM	Promoter	Location (ATG Start)
pyrG-1	*pyrG*	GCCAGCGGGTTGGTGTGCTT	TTTN	U3-AF	43–62
pyrG-2	*pyrG*	GGCGGGGCCCTACGGTCAGC	TTTN	U3-AF	151–170
pyrG-3	*pyrG*	TCATGGGGTTCGTGTCGACG	TTTN	U3-AF	632–650

#### Construction of pre-crRNA for *pyrG* gene editing in *A. aculeatus* TBRC 277

The protospacer guide sequences were designed based on the criteria used in *S. cerevisiae* [40]. Specifically, crRNA sequence targeting *pyrG* was first determined by searching for potential PAM sites (5′-TTTV-) together with a 20–23 bp protospacer sequence directly at the 3′-end of the PAM motifs using Geneious Prime ver. 2019.2.1 software [20, 61]. To ensure no off-targets, the obtained protospacer sequences were BLAST against *A. aculeatus* TBRC 277 genome sequence. The guide sequences of *pyrG* used in this study are shown in Table 2.

The U3-Af Pol III promoter and U3-Af terminator derived from *Aspergillus fumigatus* for crRNA expression was PCR amplified from pFC902 [22] by using AF U3 Prom-F and U3-TT R primers (Supplementary Table S1). The amplified PCR product was cloned into pJET1.2/Blunt vector to generate pJET1.2-U3P sgRNA plasmid, and verified by sequencing. This plasmid was then used as DNA template for the synthesis of three crRNA cassettes carrying specific Cpf1-associated protospacers (pyrG-1, pyrG-2 and pyrG-3) flanked by tRNA-Gly motif (the crRNA-tRNA-pyrG cassettes). These cassettes were individually constructed using PCR reactions with primers (Supplementary Table S1) to remove Cas9-associated sgRNA. The cassettes were used to generate Cpf1-associated crRNA cassettes which were divided into two DNA fragments each containing the appropriate homologous sequence overlaps. The corresponding purified PCR fragments were assembled into the linear *Bgl*II-cut pOK12 plasmid using Gibson assembly method [54]. Then each crRNA cassette was individually subcloned into *Bgl*II-cut pCRISPR01-FnCpf1 to generate the plasmids pCRISPR01-FnCpf1-pyrG-1, pCRISPR01-FnCpf1-pyrG-2 and pCRISPR01-FnCpf1-pyrG-3, each carrying the specific protospacer pyrG-1, pyrG-2 and pyrG-3, respectively.

#### Verification of recombinant TBRC 277 and analysis of indels

Fungal genomic DNA of randomly selected mutants was purified by Phire plant direct PCR kit (Thermo Fisher, USA) according to manufacturer’s instructions. For detection of the recombinant plasmids in TBRC 277, two pairs of primers (seq TEF-F/seq TEF-R or seq CRISPR sgRNA F-1/seq CRISPR sgRNA R-1) were employed to verify *Cpf1* gene inserts or crRNA cassettes. For detection of the genotype of uridine auxotrophic mutants, a pair of 5′-169 bp up F3/3′-148 bp down R2 primers was used to amplify *pyrG* gene, followed by DNA sequencing to determine the presence of mutations (indels) within the targeted *pyrG* loci. The CRISPR efficiency was calculated by dividing the number of mutant colonies detected by sequencing and the total number of putative mutants selected for analysis [20].

## Supplementary Information

**Additional file 1: Fig. S1.** Schematic representation of pCRISPR01 plasmid construction backbone for CRISPR/Cpf1 genome editing experiment in *A. aculeatus* TBRC 277.

**Additional file 2: Fig. S2.** Western blot detection of heterologous EGFP and Cpf1-EGFP proteins in recombinant *A. aculeatus* TBRC 277 *egfp* and *Cpf1-egfp*, respectively. (a) GenCRISPR^TM^ FnCpf1 monoclonal antibody (9H6) was used as the primary detection, 1.0 μg (Genscript, USA). (b) GFP monoclonal antibody (C163) was used for the primary detection, 3 μg (Thermo Fisher, USA). All samples were treated by IgG-AP as the secondary antibody for detection, 2 μg. Lane 1, crude protein of *A. aculeatus* recombinant (*Cpf1-egfp*). Lane 2, crude protein of recombinant *A. aculeatus* (*egfp*), Lane 3, crude protein of *A. aculeatus* TBRC 277 wild-type (control). Each lane was loaded with 20-μg protein. Anti-GFP (Roche) and FnCpf1 (Genscript, USA) antibodies were used as the primary (monoclonal) antibodies, anti IgG-conjugated AP was used for secondary antibody. M: PageRule Plus Prestained Protein Ladder (Thermo Fisher, USA).

**Additional file 3: Fig. S3.** The functional plasmid construction of CRISPR/Cpf1 system. The plasmid consists of Cpf1 nuclease gene and crRNA-pyrG cassettes for targeted genome modification in *A. aculeatus* TBRC 277. (a) pCRISPR/Cpf1-pyrG plasmid, crRNA is expressed under control of U3-AF pol. III promoter, (b) Guide RNA cassette, crRNA, consists of 19-bp direct repeats (DR) to form a stem-loop structure, as well as 20-bp *pyrG*-targeted protospacers.

**Additional file 4: Fig. S4.** Transformation efficiency of *A. aculeatus* TBRC 277 in the presence or absence of Cpf1 endonuclease containing plasmids. To investigate the toxicity of Cpf1 on the *A. aculeatus* host, 10-μg DNA of each plasmids were independently transformed into TBRC 277 protoplast**.** The number of transformants were recovered from minimal medium (MM+Czapek-Dox+bleomycin+sorbitol) supplemented with Uri/Ura. Protoplast transformed with empty vector**,** pCRISPR01**,** has no *FnCpf1* gene (dark grey); protoplast transformed with *FnCpf1*-containing plasmid**,** pCRISPR01-FnCpf1 (light-grey); protoplast transformed with *FnCpf1* and crRNA-pyrGs**,** pCRISPR01-FnCpf1-pyrGs (pyrG-1, pyrG-2, or pyrG-3) (white). The graph shows the means and standard deviation (SD) from two independent experiments.

**Additional file 5: Table S1.** CRISPR/Cpf1 gene editing efficiency targeting pyrGof A. aculeatus TBRC 277.

**Additional file 6: Table S2.** List of primers used in this study.

**Additional file 7: Table S3.** The sequence of *FnCpf1, eGFP*, and pCRISPR01 expression vector.

## Data Availability

All data generated or analysed during this study are included in this published article. The open reading frame of *pyrG* gene sequence was submitted to NCBI sequence database under the accession number MN364695. Plasmids are available upon reasonable request as well as on the TBRC website: https://www.tbrcnetwork.org.

## Abbreviations

5-FOA: 5-fluoroorotic acid
Cas: CRISPR-associated proteins
CRISPR: Clustered regularly interspaced short palindromic repeats
crRNA: CRISPR RNA or guide RNA or gRNA
eGFP: Enhanced green fluorescent protein
MM: Minimal medium
NHEJ: Non-homologous end-joining
NLS: Nuclear localization signal
PAM: Protospacer adjacent motif

## References

[1] Suwannarangsee S, Arnthong J, Eurwilaichitr L, Champreda V (2014). Production and characterization of multi-polysaccharide degrading enzymes from *Aspergillus aculeatus* BCC199 for saccharification of agricultural residues. J Microbiol Biotechnol.

[2] Ward OP (2012). Production of recombinant proteins by filamentous fungi. Biotechnol Adv.

[3] Nielsen JC, Grijseels S, Prigent S, Ji B, Dainat J, Nielsen KF (2017). Global analysis of biosynthetic gene clusters reveals vast potential of secondary metabolite production in Penicillium species. Nat Microbiol.

[4] de Vries RP, Riley R, Wiebenga A, Aguilar-Osorio G, Amillis S, Uchima CA (2017). Comparative genomics reveals high biological diversity and specific adaptations in the industrially and medically important fungal genus Aspergillus. Genome Biol.

[5] Li Y, Hu X, Sang J, Zhang Y, Zhang H, Lu F (2018). An acid-stable beta-glucosidase from *Aspergillus aculeatus*: gene expression, biochemical characterization and molecular dynamics simulation. Int J biol Macromol.

[6] Klein-Marcuschamer D, Oleskowicz-Popiel P, Simmons BA, Blanch HW (2012). The challenge of enzyme cost in the production of lignocellulosic biofuels. Biotechnol Bioeng.

[7] Sarkari P, Marx H, Blumhoff ML, Mattanovich D, Sauer M, Steiger MG (2017). An efficient tool for metabolic pathway construction and gene integration for *Aspergillus niger*. Bioresour Technol.

[8] Meyer V (2008). Genetic engineering of filamentous fungi — Progress, obstacles and future trends. Biotechnol Advances.

[9] Leynaud-Kieffer LMC, Curran SC, Kim I, Magnuson JK, Gladden JM, Baker SE (2019). A new approach to Cas9-based genome editing in *Aspergillus niger* that is precise, efficient and selectable. PloS one.

[10] Kjærbølling I, Vesth TC, Frisvad JC, Nybo JL, Theobald S, Kuo A, et al. Linking secondary metabolites to gene clusters through genome sequencing of six diverse Aspergillus species. Proc Nat Acad Sci USA. 2018;115(4):E753-EE61. doi: 10.1073/pnas.1715954115. 10.1073/pnas.1715954115 PMC5789934 29317534

[11] Besser J, Carleton HA, Gerner-Smidt P, Lindsey RL, Trees E (2018). Next-generation sequencing technologies and their application to the study and control of bacterial infections. Clin Microbiol Infect.

[12] Shi TQ, Liu GN, Ji RY, Shi K, Song P, Ren LJ (2017). CRISPR/Cas9-based genome editing of the filamentous fungi: the state of the art. Appl Microbiol Biotechnol.

[13] Hsu PD, Lander ES, Zhang F (2014). Development and applications of CRISPR-Cas9 for genome engineering. Cell.

[14] Cong L, Ran FA, Cox D, Lin S, Barretto R, Habib N (2013). Multiplex genome engineering using CRISPR/Cas systems. Science.

[15] Jinek M, Chylinski K, Fonfara I, Hauer M, Doudna JA, Charpentier E (2012). A programmable dual-RNA-guided DNA endonuclease in adaptive bacterial immunity. Science.

[16] Fuller KK, Chen S, Loros JJ, Dunlap JC (2015). Development of the CRISPR/Cas9 system for targeted gene disruption in *Aspergillus fumigatus*. Eukaryotic cell.

[17] Katayama T, Tanaka Y, Okabe T, Nakamura H, Fujii W, Kitamoto K (2016). Development of a genome editing technique using the CRISPR/Cas9 system in the industrial filamentous fungus *Aspergillus oryzae*. Biotechnol Lett.

[18] Dong H, Zheng J, Yu D, Wang B, Pan L (2019). Efficient genome editing in *Aspergillus niger* with an improved recyclable CRISPR-HDR toolbox and its application in introducing multiple copies of heterologous genes. J Microbiol Methods.

[19] Kuivanen J, Wang YMJ, Richard P (2016). Engineering *Aspergillus niger* for galactaric acid production: elimination of galactaric acid catabolism by using RNA sequencing and CRISPR/Cas9. Microbial Cell Factories.

[20] Song L, Ouedraogo J-P, Kolbusz M, Nguyen TTM, Tsang A (2018). Efficient genome editing using tRNA promoter-driven CRISPR/Cas9 gRNA in *Aspergillus niger*. PloS one.

[21] Nodvig CS, Hoof JB, Kogle ME, Jarczynska ZD, Lehmbeck J, Klitgaard DK (2018). Efficient oligo nucleotide mediated CRISPR-Cas9 gene editing in Aspergilli. Fungal genet biol.

[22] Nodvig CS, Nielsen JB, Kogle ME, Mortensen UH (2015). A CRISPR-Cas9 system for genetic engineering of filamentous Fungi. PLoS one.

[23] Liu R, Chen L, Jiang Y, Zhou Z, Zou G (2015). Efficient genome editing in filamentous fungus *Trichoderma reesei* using the CRISPR/Cas9 system. Cell Discov.

[24] Matsu-Ura T, Baek M, Kwon J, Hong C (2015). Efficient gene editing in *Neurospora crassa* with CRISPR technology. Fungal Biol Biotechnol.

[25] Qin H, Xiao H, Zou G, Zhou Z, Zhong J-J (2017). CRISPR-Cas9 assisted gene disruption in the higher fungus Ganoderma species. Process Biochem.

[26] Liu Q, Gao R, Li J, Lin L, Zhao J, Sun W, et al. Development of a genome-editing CRISPR/Cas9 system in thermophilic fungal Myceliophthora species and its application to hyper-cellulase production strain engineering. Biotechnol biofuels. 2017;10(1). 10.1186/s13068-016-0693-9. 10.1186/s13068-016-0693-9 PMC5209885 28053662

[27] Zetsche B, Gootenberg JS, Abudayyeh OO, Slaymaker IM, Makarova KS, Essletzbichler P (2015). Cpf1 is a single RNA-guided endonuclease of a class 2 CRISPR-Cas system. Cell.

[28] Koonin EV, Makarova KS, Zhang F (2017). Diversity, classification and evolution of CRISPR-Cas systems. Curr Opin Microbiol.

[29] Zetsche B, Heidenreich M, Mohanraju P, Fedorova I, Kneppers J, DeGennaro EM (2017). Multiplex gene editing by CRISPR-Cpf1 using a single crRNA array. Nat Biotechnol.

[30] Wang M, Mao Y, Lu Y, Tao X, Zhu JK (2017). Multiplex gene editing in Rice using the CRISPR-Cpf1 system. Mol Plant.

[31] Fonfara I, Richter H, Bratovic M, Le Rhun A, Charpentier E (2016). The CRISPR-associated DNA-cleaving enzyme Cpf1 also processes precursor CRISPR RNA. Nature.

[32] Deng H, Gao R, Liao X, Cai Y (2017). CRISPR system in filamentous fungi: current achievements and future directions. Gene..

[33] White RJ (2011). Transcription by RNA polymerase III: more complex than we thought. Nat Rev Genet.

[34] Gao Y, Zhao Y (2014). Self-processing of ribozyme-flanked RNAs into guide RNAs in vitro and in vivo for CRISPR-mediated genome editing. J Integr plant biol.

[35] Xie K, Minkenberg B, Yang Y (2015). Boosting CRISPR/Cas9 multiplex editing capability with the endogenous tRNA-processing system. Proceedings of the National Academy of Sciences.

[36] Vanegas KG, Jarczynska ZD, Strucko T, Mortensen UH (2019). Cpf1 enables fast and efficient genome editing in Aspergilli. Fungal biol Biotechnol.

[37] Jiménez A, Hoff B, Revuelta JL (2020). Multiplex genome editing in *Ashbya gossypii* using CRISPR-Cpf1. New biotechnology.

[38] Liu Q, Zhang Y, Li F, Li J, Sun W, Tian C (2019). Upgrading of efficient and scalable CRISPR-Cas-mediated technology for genetic engineering in thermophilic fungus *Myceliophthora thermophila*. Biotechnology for biofuels.

[39] Li ZH, Liu M, Lyu XM, Wang FQ, Wei DZ (2018). CRISPR/Cpf1 facilitated large fragment deletion in *Saccharomyces cerevisiae*. J basic Microbiol.

[40] Swiat MA, Dashko S, den Ridder M, Wijsman M, van der Oost J, Daran JM (2017). FnCpf1: a novel and efficient genome editing tool for *Saccharomyces cerevisiae*. Nucleic acids res.

[41] Verwaal R, Buiting-Wiessenhaan N, Dalhuijsen S, Roubos JA (2018). CRISPR/Cpf1 enables fast and simple genome editing of *Saccharomyces cerevisiae*. Yeast.

[42] Boeke JD, LaCroute F, Fink GR (1984). A positive selection for mutants lacking orotidine-5′-phosphate decarboxylase activity in yeast: 5-fluoro-orotic acid resistance. Mol Gen Genet.

[43] Ling SO, Storms R, Zheng Y, Rodzi MR, Mahadi NM, Illias RM (2013). Development of a pyrG mutant of *Aspergillus oryzae* strain S1 as a host for the production of heterologous proteins. Scientific World J.

[44] d'Enfert C (1996). Selection of multiple disruption events in *Aspergillus fumigatus* using the orotidine-5′-decarboxylase gene, pyrG, as a unique transformation marker. Curr Genet.

[45] Weidner G, d'Enfert C, Koch A, Mol PC, Brakhage AA (1998). Development of a homologous transformation system for the human pathogenic fungus *Aspergillus fumigatus* based on the pyrG gene encoding orotidine 5′-monophosphate decarboxylase. Curr Genet.

[46] Kim H, Kim ST, Ryu J, Kang BC, Kim JS, Kim SG (2017). CRISPR/Cpf1-mediated DNA-free plant genome editing. Nat Commun.

[47] Swarts DC, Jinek M. Cas9 versus Cas12a/Cpf1: structure-function comparisons and implications for genome editing. Wiley Interdiscip rev RNA. 2018:e1481. 10.1002/wrna.1481. 10.1002/wrna.1481 29790280

[48] Jöchl C, Rederstorff M, Hertel J, Stadler PF, Hofacker IL, Schrettl M (2008). Small ncRNA transcriptome analysis from *Aspergillus fumigatus* suggests a novel mechanism for regulation of protein synthesis. Nucleic Acids Res.

[49] Swarts DC, van der Oost J, Jinek M (2017). Structural basis for guide RNA processing and seed-dependent DNA targeting by CRISPR-Cas12a. Mol Cell.

[50] Jiang Y, Qian F, Yang J, Liu Y, Dong F, Xu C (2017). CRISPR-Cpf1 assisted genome editing of *Corynebacterium glutamicum*. Nat Commun.

[51] Ding D, Chen K, Chen Y, Li H, Xie K (2018). Engineering introns to express RNA guides for Cas9- and Cpf1-mediated multiplex genome editing. Mol Plant.

[52] Arentshorst M, Ram AFJ, Meyer V, Bolton MD, Thomma BPHJ (2012). Using non-homologous end-joining-deficient strains for functional gene analyses in filamentous Fungi. Plant fungal pathogens: methods and protocols.

[53] Vieira J, Messing J. New pUC-derived cloning vectors with different selectable markers and DNA replication origins. Gene. 100:1991, 189–4. 10.1016/0378-1119(91)90365-I. 10.1016/0378-1119(91)90365-i 1905257

[54] Gibson DG, Young L, Chuang RY, Venter JC, Hutchison CA, Smith HO (2009). Enzymatic assembly of DNA molecules up to several hundred kilobases. Nat methods.

[55] Johnstone IL, Hughes SG, Clutterbuck AJ (1985). Cloning an *Aspergillus nidulans* developmental gene by transformation. EMBO J.

[56] Penttilä M, Nevalainen H, Rättö M, Salminen E, Knowles J (1987). A versatile transformation system for the cellulolytic filamentous fungus *Trichoderma reesei*. Gene.

[57] Berger H, Pachlinger R, Morozov I, Goller S, Narendja F, Caddick M (2006). The GATA factor AreA regulates localization and in vivo binding site occupancy of the nitrate activator NirA. Mol Microbiol.

[58] Vaknin Y, Hillmann F, Iannitti R, Ben Baruch N, Sandovsky-Losica H, Shadkchan Y (2016). Identification and characterization of a novel *Aspergillus fumigatus* rhomboid family putative protease, RbdA, involved in hypoxia sensing and virulence. Infect Immun.

[59] Zhang C, Meng X, Wei X, Lu L (2016). Highly efficient CRISPR mutagenesis by microhomology-mediated end joining in *Aspergillus fumigatus*. Fungal Genet Biol.

[60] Bradford MM (1976). A rapid and sensitive method for the quantitation of microgram quantities of protein utilizing the principle of protein-dye binding. Analytical biochem.

[61] Kearse M, Moir R, Wilson A, Stones-Havas S, Cheung M, Sturrock S (2012). Geneious basic: an integrated and extendable desktop software platform for the organization and analysis of sequence data. Bioinformatics.

