# Supplementary material for: Development of a CRISPR/Cpf1 system for targeted gene disruption in Aspergillus aculeatus TBRC 277
Source: BMC Biotechnol. 2021 Feb 11;21:15. doi: 10.1186/s12896-021-00669-8 (PMC7879532; doi:10.1186/s12896-021-00669-8)
Supplement: Supplementary file 1 — Additional file 1: Fig. S1. Schematic representation of pCRISPR01 plasmid construction backbone for CRISPR/Cpf1 genome editing experiment in A. aculeatus TBRC 277. [file 12896_2021_669_MOESM1_ESM.docx]

**Fig S1.** Schematic representation of pCRISPR01 plasmid construction backbone for CRISPR/Cpf1 genome editing experiment in *A. aculeatus* TBRC 277.
