# Supplementary material for: Development of a CRISPR/Cpf1 system for targeted gene disruption in Aspergillus aculeatus TBRC 277
Source: BMC Biotechnol. 2021 Feb 11;21:15. doi: 10.1186/s12896-021-00669-8 (PMC7879532; doi:10.1186/s12896-021-00669-8)
Supplement: Supplementary file 2 — Additional file 2: Fig. S2. Western blot detection of heterologous EGFP and Cpf1-EGFP proteins in recombinant A. aculeatus TBRC 277 egfp and Cpf1-egfp, respectively. (a) GenCRISPRTM FnCpf1 monoclonal antibody (9H6) was used as the primary detection, 1.0 μg (Genscript, USA). (b) GFP monoclonal antibody (C163) was used for the primary detection, 3 μg (Thermo Fisher, USA). All samples were treated by IgG-AP as the secondary antibody for detection, 2 μg. Lane 1, crude protein of A. aculeatus recombinant (Cpf1-egfp). Lane 2, crude protein of recombinant A. aculeatus (egfp), Lane 3, crude protein of A. aculeatus TBRC 277 wild-type (control). Each lane was loaded with 20-μg protein. Anti-GFP (Roche) and FnCpf1 (Genscript, USA) antibodies were used as the primary (monoclonal) antibodies, anti IgG-conjugated AP was used for secondary antibody. M: PageRule Plus Prestained Protein Ladder (Thermo Fisher, USA). [file 12896_2021_669_MOESM2_ESM.docx]

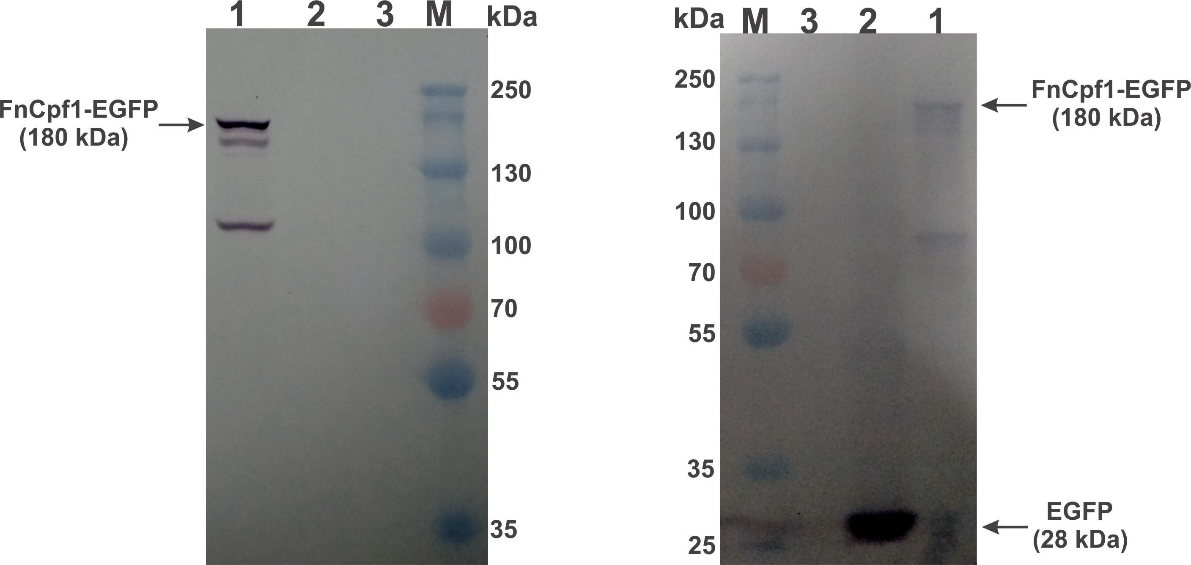


**(a) (b)**

**Fig S2.** Western blot detection of heterologous EGFP and Cpf1-EGFP proteins in recombinant *A. aculeatus* TBRC 277 *egfp* and *Cpf1-egfp*, respectively. **(a)** GenCRISPRᵀᴹ FnCpf1 monoclonal antibody (9H6) was used as the primary detection, 1.0 µg (Genscript, USA). **(b)** GFP monoclonal antibody (C163) was used for the primary detection, 3 µg (Thermo Fisher, USA). All samples were treated by IgG-AP as the secondary antibody for detection, 2 µg. Lane 1, crude protein of *A. aculeatus* recombinant (*Cpf1-egfp*). Lane 2, crude protein of recombinant *A. aculeatus* (*egfp*), Lane 3: Crude protein of *A. aculeatus* TBRC 277 wild-type (control). Each lanes was loaded with 20-µg protein. Anti-GFP (Roche) and FnCpf1 (Genscript, USA) antibody was used as the primary (monoclonal) antibody, anti IgG-conjugated AP was used for secondary antibody. M: PageRuler™ Plus Prestained Protein Ladder (Thermo Fisher, USA). The gels were processed independently.
