# Supplementary material for: Development of a CRISPR/Cpf1 system for targeted gene disruption in Aspergillus aculeatus TBRC 277
Source: BMC Biotechnol. 2021 Feb 11;21:15. doi: 10.1186/s12896-021-00669-8 (PMC7879532; doi:10.1186/s12896-021-00669-8)
Supplement: Supplementary file 3 — Additional file 3: Fig. S3. The functional plasmid construction of CRISPR/Cpf1 system. The plasmid consists of Cpf1 nuclease gene and crRNA-pyrG cassettes for targeted genome modification in A. aculeatus TBRC 277. (a) pCRISPR/Cpf1-pyrG plasmid, crRNA is expressed under control of U3-AF pol. III promoter, (b) Guide RNA cassette, crRNA, consists of 19-bp direct repeats (DR) to form a stem-loop structure, as well as 20-bp pyrG-targeted protospacers. [file 12896_2021_669_MOESM3_ESM.docx]

**(a)**

**(b)**

**Fig S3.** The functional plasmid construction of CRISPR/Cpf1 system. The plasmid consists of Cpf1 nuclease gene and crRNA-pyrG cassettes for targeted genome modification in *A. aculeatus* TBRC 277. **(a)** pCRISPR/Cpf1-pyrG plasmid, crRNA is expressed under control of U3-AF pol. III promoter, **(b)** Guide RNA cassette, crRNA, consists of 19-bp direct repeats (DR) to form a stem-loop structure, as well as 20-bp *pyrG*-targeted protospacers.
