# Supplementary material for: Development of a CRISPR/Cpf1 system for targeted gene disruption in Aspergillus aculeatus TBRC 277
Source: BMC Biotechnol. 2021 Feb 11;21:15. doi: 10.1186/s12896-021-00669-8 (PMC7879532; doi:10.1186/s12896-021-00669-8)
Supplement: Supplementary file 4 — Additional file 4: Fig. S4. Transformation efficiency of A. aculeatus TBRC 277 in the presence or absence of Cpf1 endonuclease containing plasmids. To investigate the toxicity of Cpf1 on the A. aculeatus host, 10-μg DNA of each plasmids were independently transformed into TBRC 277 protoplast. The number of transformants were recovered from minimal medium (MM+Czapek-Dox+bleomycin+sorbitol) supplemented with Uri/Ura. Protoplast transformed with empty vector, pCRISPR01, has no FnCpf1 gene (dark grey); protoplast transformed with FnCpf1-containing plasmid, pCRISPR01-FnCpf1 (light-grey); protoplast transformed with FnCpf1 and crRNA-pyrGs, pCRISPR01-FnCpf1-pyrGs (pyrG-1, pyrG-2, or pyrG-3) (white). The graph shows the means and standard deviation (SD) from two independent experiments. [file 12896_2021_669_MOESM4_ESM.zip › additional file 4 figure s4.docx]

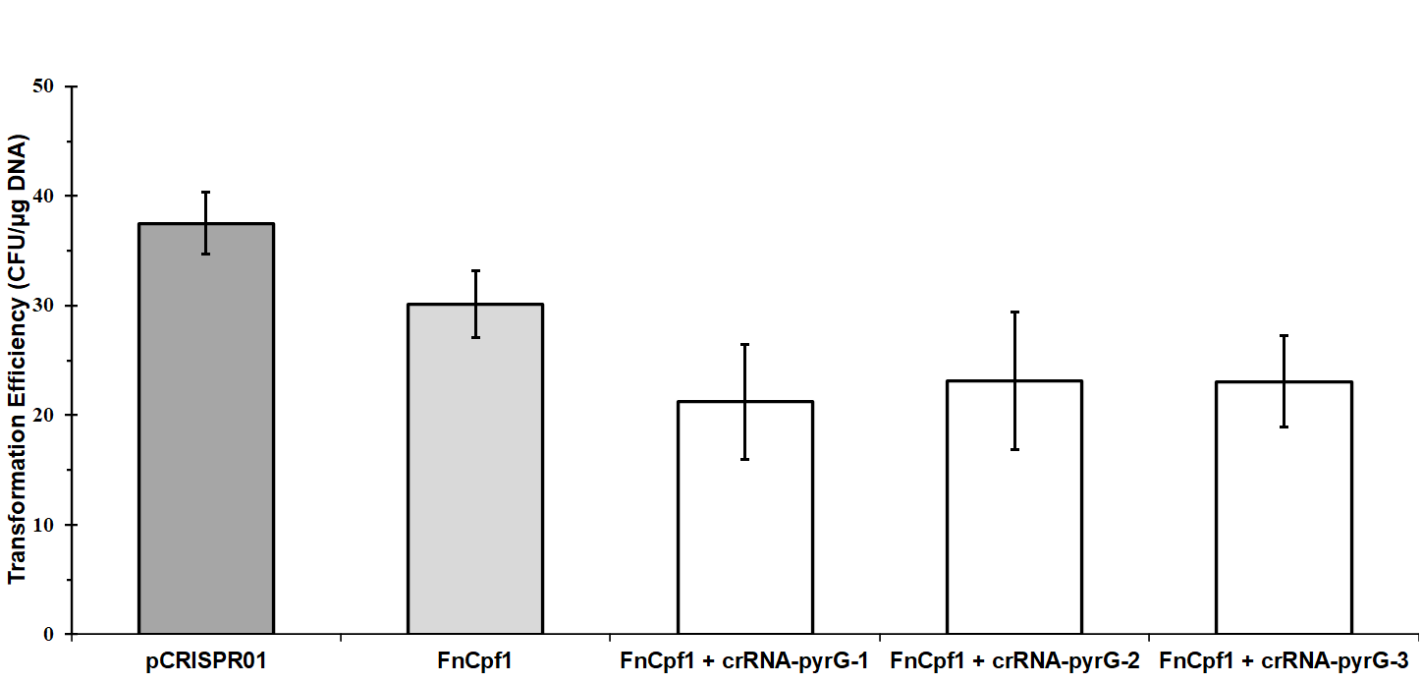


**Fig S4**. Transformation efficiency of *A. aculeatus* TBRC 277 in the presence or absence of Cpf1 endonuclease containing plasmids. To investigate the toxicity of Cpf1 on the *A. aculeatus* host, 10-µg DNA of each plasmids were independently transformed into TBRC 277 protoplast. The number of transformants were recovered from minimal medium (MM+Czapek-Dox+bleomycin+sorbitol) supplemented with Uri/Ura. Protoplast transformed with empty vector, pCRISPR01, has no *FnCpf1* gene (dark grey); protoplast transformed with *FnCpf1*-containing plasmid, pCRISPR01-FnCpf1 (light-grey); protoplast transformed with *FnCpf1* and crRNA-pyrGs, pCRISPR01-FnCpf1-pyrGs (pyrG-1, pyrG-2, or pyrG-3) (white). The graph shows the means and standard deviation (SD) from two independent experiments. (DOCX 67 kb)
