# Supplementary material for: Development of a CRISPR/Cpf1 system for targeted gene disruption in Aspergillus aculeatus TBRC 277
Source: BMC Biotechnol. 2021 Feb 11;21:15. doi: 10.1186/s12896-021-00669-8 (PMC7879532; doi:10.1186/s12896-021-00669-8)
Supplement: Supplementary file 4 — Additional file 4: Fig. S4. Transformation efficiency of A. aculeatus TBRC 277 in the presence or absence of Cpf1 endonuclease containing plasmids. To investigate the toxicity of Cpf1 on the A. aculeatus host, 10-μg DNA of each plasmids were independently transformed into TBRC 277 protoplast. The number of transformants were recovered from minimal medium (MM+Czapek-Dox+bleomycin+sorbitol) supplemented with Uri/Ura. Protoplast transformed with empty vector, pCRISPR01, has no FnCpf1 gene (dark grey); protoplast transformed with FnCpf1-containing plasmid, pCRISPR01-FnCpf1 (light-grey); protoplast transformed with FnCpf1 and crRNA-pyrGs, pCRISPR01-FnCpf1-pyrGs (pyrG-1, pyrG-2, or pyrG-3) (white). The graph shows the means and standard deviation (SD) from two independent experiments. [file 12896_2021_669_MOESM4_ESM.zip › additional file 4 table s1.docx.docx]

**Table S1.** **CRISPR/Cpf1** **gene editing efficiency targeting *pyrG* of *A. aculeatus*** **TBRC 277**

| Plasmids | Selected Transformants | Mutant auxotroph Screened on 5-FOA | *pyrG* sequencing of selected mutants (%) |
| --- | --- | --- | --- |
| - | 0 | 0 | 0 |
| pCRISPR01-FnCpf1 | 20 | 0 | 0 |
| pCRISPR01-FnCpf1-pyrG-1 | 20 | 10/10 (100%) | 3/3 (100%) |
| pCRISPR01-FnCpf1-pyrG-2 | 20 | 10/10 (100%) | 3/3 (100%) |
| pCRISPR01-FnCpf1-pyrG-3 | 20 | 10/10 (100%) | 3/3 (100%) |
