# Supplementary material for: Development of a CRISPR/Cpf1 system for targeted gene disruption in Aspergillus aculeatus TBRC 277
Source: BMC Biotechnol. 2021 Feb 11;21:15. doi: 10.1186/s12896-021-00669-8 (PMC7879532; doi:10.1186/s12896-021-00669-8)
Supplement: Supplementary file 6 — Additional file 6: Table S2. List of primers used in this study. [file 12896_2021_669_MOESM6_ESM.docx]

**Table S2. List of primers used in this study**

| **Code** | **Primer name** | **Sequence^A^** | **Purpose** |
| --- | --- | --- | --- |
| **Oligonucleotide primers for construction CRISPR/Cpf1 plasmid bacbone** | | |  |
| 001 | pFC333-TT-NcoI-F | GAATTCCCATGGGGTACCGTTTAAACGCGGACATTCGATTTATGCCGTTATGACTTCC | Cloning TEF TT+MCS |
| 002 | pFC333-TT-BamHI-R | CATTTTTAATGTCTTGAATCGCGCATTGGATCCTGCCC | Cloning TEF TT+MCS |
| 003 | FnCpf1-Nco-I-F | CCATCCATGGCCATCTACCAGGAGTTCGTCAACAAGTAC | Cloning FnCpf1+ SV40-NLS gene |
| 004 | FnCpf1-PmeI-I-R | GATCCGATGTTTAAACCTACACCTTGCGCTTC | Cloning FnCpf1+ SV40-NLS gene |
| 005 | FnCpf1-Kpn-R | CTTCTTGGTACCGTCGGCGCGGCTGTTGTTGCGGTTC | Cloning FnCpf1 gene fusion |
| 006 | EGFP-NK-F | CTGGTCCCATGGGGTACCATGGTCAGCAAGGGCGAGGAGCTGTTC | Cloning EGFP+SV40-NLS gene |
| 007 | EGFP-SV40-Pme-R | GGATCCGTTTAAACCTACACTTTGCGCTTCTTCTTCGGAGGCTTGTACAGCTCGTCCA  TGCCC | Cloning EGFP+SV40-NLS gene |
| 008 | KanR-Bgl2-F | AAAACGAAGATCTGAAGATCATCTTATTAAGGGGTCTGACGCTCAGTGG | Cloning of kanamycin cassette |
| 009 | KanR-R | AGCGGATAACAATTTCACACAGG | Cloning of kanamycin cassette |
| **Oligonucleotide primers for crRNA construction targeting *pyrG* locus in *A. aculeatus*** | | | |
| 010 | AF-U3 Prom F | CACGTGACTAGTAGATCTGATCACATAGATGCTCGGTTGACAGG | Cloning sgRNA-cassette from pFC902 |
| 011 | U3-TT R | CACGTGACTAGTAGATCTggatccACCCTGAGAAGATAGATGTGAATGTGTG | Cloning sgRNA-cassette from pFC902 |
| 012 | GA AF U3 Prom-F | tgtaatacgactcactatagggcgaattggggatcgatccACTAGTAGATCTGA  TCACATAGATGCTCGGTTGACAGG | Amplification 5’ *U3 promoter* |
| 013 | GA U3-TT R | acaatttcacacaggaaacagctatgaccatgattacgccACTAGTAGATCTggatcc  ACCCTGAGAAGATAGATGTGAATGTGTG | Amplification 3’ *U3 terminator* |
| 014 | sgRNA pyrG-1 F | TCTTGTAGATGCCAGCGGGTTGGTGTGCTTTTTTGCATCATTGGTCTAGTGGTAGAAT  TC | U3-AF crRNA-pyrG1 construction |
| 015 | sgRNA pyrG-1 R | ACCCGCTGGCATCTACAAGAGTAGAAATTATGCATCATCCGTGAATCGAACAC | U3-AF crRNA-pyrG1 construction |
| 016 | sgRNA pyrG-2 F | TCTTGTAGATTCATGGGGTTCGTGTCGACGTTTTGCATCATTGGTCTAGTGGTAGAA  TTC | U3-AF crRNA-pyrG2 construction |
| 017 | sgRNA pyrG-2 R | AACCCCATGAATCTACAAGAGTAGAAATTATGCATCATCCGTGAATCGAACAC | U3-AF crRNA-pyrG2 construction |
| 018 | sgRNA pyrG-3 F | TCTTGTAGATGGCGGGGCCCTACGGTCAGCGAGTTTTGCATCATTGGTCTAG  TGGTAGAATTC | U3-AF crRNA-pyrG3 construction |
| 019 | sgRNA pyrG-3 R | CCCCGCCATCTACAAGAGTAGAAATTATGCATCATCCGTGAATCGAACAC | U3-AF crRNA-pyrG3 construction |
|  |  |  |  |
| **Primers for diagnostic PCR and sequencing** | | |  |
| 020 | Seq pFC333-TEF F | GCCCAAGGTGGGAGTCTAGGAG | Sequencing of Insert in pCRISPR01 |
| 021 | Seq pFC333-TEF R | CGCCTGGACGACTAAACCAAAATAGG | Sequencing of Insert in pCRISPR01 |
| 022 | seq pJET1.2 F | CGACTCACTATAGGGAGAGCGGC | Sequencing of insert in pJET1.2B |
| 023 | seq pJET1.2 R | AAGAACATCGATTTTCCATGGCAG | Sequencing of insert in pJET1.2B |
| 024 | M13 F | CCCAGTCACGACGTTGTAAAACG | Sequencing of insert in pOK12 |
| 025 | M13-R | AGCGGATAACAATTTCACACAGG | Sequencing of insert in pOK12 |
| 026 | Seq FnCpf1 1 | CCAGAACCTGATCGACGCCAAG | FnCpf1 complete CDS sequencing |
| 027 | Seq FnCpf1 2 | CGAGGAGAAGTCGATCAAGGAGACC | FnCpf1 complete CDS sequencing |
| 028 | Seq FnCpf1 3 | CGGCTGGGACAAGAACAAGGAG | FnCpf1 complete CDS sequencing |
| 029 | Seq FnCpf1 4 | GAAGAAGGAGTCGGTGTTCGAGTACG | FnCpf1 complete CDS sequencing |
| 030 | pyrG-F | ATGTCTTCCAAGTCGCAATTGACCTAC | *pyrG* gene amplification |
| 031 | pyrG-R | TTAGTTTCCGCTAACACGGGCCTGGTAC | *pyrG* gene amplification |
| 032 | seq CRISPR sgRNA F-1 | TTTTGAGACACAACGTGGCTTTCCC | Sequencing crRNA cassette |
| 033 | seq CRISPR sgRNA R-1 | GCTCCTTTGCTTTTCCCGAACTTGG | Sequencing crRNA cassette |
|  |  |  |  |
| Primers for sequencing pyrG mutants | | | |
| 034 | 5'-169bp up F3 | TCTTCTTTTCGTCTATACCCCGCC | Sequencing of pyrG 5’-end |
| 035 | 3'-148 bp down R2 | GGCTGACTCAACGATCTTATCTTTCCTGCAGG | Sequencing of pyrG 3’-end |
