# Supplementary material for: Development of a CRISPR/Cpf1 system for targeted gene disruption in Aspergillus aculeatus TBRC 277
Source: BMC Biotechnol. 2021 Feb 11;21:15. doi: 10.1186/s12896-021-00669-8 (PMC7879532; doi:10.1186/s12896-021-00669-8)
Supplement: Supplementary file 7 — Additional file 7: Table S3. The sequence of FnCpf1, eGFP, and pCRISPR01 expression vector. [file 12896_2021_669_MOESM7_ESM.docx]

**Table S3. The sequence of *FnCpf1,* eGFP, and pCRISPR01 expression vector.**

| ***FnCpf1+* SV40 NLS (3,936 bp)** |
| --- |
| ATGGCCATCTACCAGGAGTTCGTCAACAAGTACAGCCTGTCCAAGACCCTGCGCTTCGAGCTGATCCCGCAGGGCAAGACCCTGGAGAACATCAAGGCTCGCGGCCTGATCCTGGACGACGAGAAGCGCGCCAAGGACTACAAGAAGGCCAAGCAGATCATCGACAAGTACCACCAGTTCTTCATCGAGGAGATCCTGAGCAGCGTCTGCATCTCGGAGGACCTGCTCCAGAACTACAGCGACGTGTACTTCAAGCTGAAGAAGTCGGACGACGACAACCTCCAGAAGGACTTCAAGAGCGCCAAGGACACCATCAAGAAGCAGATCAGCGAGTACATCAAGGACTCCGAGAAGTTCAAGAACCTGTTCAACCAGAACCTGATCGACGCCAAGAAGGGCCAGGAGTCCGACCTGATCCTGTGGCTGAAGCAGTCGAAGGACAACGGCATCGAGCTGTTCAAGGCCAACTCGGACATCACCGACATCGACGAGGCCCTGGAGATCATCAAGAGCTTCAAGGGCTGGACCACCTACTTCAAGGGCTTCCACGAGAACCGCAAGAACGTCTACAGCAGCAACGACATCCCGACCAGCATCATCTACCGCATCGTGGACGACAACCTGCCCAAGTTCCTGGAGAACAAGGCCAAGTACGAGTCCCTGAAGGACAAGGCCCCCGAGGCCATCAACTACGAGCAGATCAAGAAGGACCTGGCCGAGGAGCTGACCTTCGACATCGACTACAAGACCTCCGAGGTCAACCAGCGCGTGTTCTCGCTGGACGAGGTCTTCGAGATCGCCAACTTCAACAACTACCTGAACCAGTCGGGCATCACCAAGTTCAACACCATCATCGGCGGCAAGTTCGTGAACGGCGAGAACACCAAGCGCAAGGGCATCAACGAGTACATCAACCTGTACAGCCAGCAGATCAACGACAAGACCCTGAAGAAGTACAAGATGAGCGTCCTGTTCAAGCAGATCCTGTCCGACACCGAGTCCAAGAGCTTCGTGATCGACAAGCTGGAGGACGACTCGGACGTCGTGACCACCATGCAGAGCTTCTACGAGCAGATCGCCGCCTTCAAGACCGTCGAGGAGAAGTCGATCAAGGAGACCCTGAGCCTGCTGTTCGACGACCTGAAGGCCCAGAAGCTGGACCTGTCGAAGATTTACTTCAAGAACGACAAGTCCCTGACCGACCTGTCGCAGCAGGTGTTCGACGACTACAGCGTCATCGGCACCGCCGTGCTGGAGTACATCACCCAGCAGATCGCCCCGAAGAACCTGGACAACCCCTCGAAGAAGGAGCAGGAGCTGATCGCCAAGAAGACCGAGAAGGCCAAGTACCTGAGCCTGGAGACCATCAAGCTGGCCCTGGAGGAGTTCAACAAGCACCGCGACATCGACAAGCAGTGCCGCTTCGAGGAGATCCTGGCCAACTTCGCCGCCATCCCGATGATCTTCGACGAGATCGCCCAGAACAAGGACAACCTGGCCCAGATCAGCATCAAGTACCAGAACCAGGGCAAGAAGGACCTGCTCCAGGCCAGCGCCGAGGACGACGTCAAGGCCATCAAGGACCTGCTGGACCAGACCAACAACCTGCTGCACAAGCTGAAGATTTTCCACATCTCCCAGTCGGAGGACAAGGCCAACATCCTGGACAAGGACGAGCACTTCTACCTGGTCTTCGAGGAGTGCTACTTCGAGCTGGCCAACATCGTGCCGCTGTACAACAAGATCCGCAACTACATCACCCAGAAGCCCTACTCCGACGAGAAGTTCAAGCTGAACTTCGAGAACTCGACCCTGGCCAACGGCTGGGACAAGAACAAGGAGCCCGACAACACCGCCATCCTGTTCATCAAGGACGACAAGTACTACCTGGGCGTGATGAACAAGAAGAACAACAAGATTTTCGACGACAAGGCCATCAAGGAGAACAAGGGCGAGGGCTACAAGAAGATCGTCTACAAGCTGCTGCCGGGCGCCAACAAGATGCTGCCCAAGGTGTTCTTCTCCGCCAAGTCGATCAAGTTCTACAACCCGTCCGAGGACATCCTGCGCATCCGCAACCACTCCACCCACACCAAGAACGGCTCGCCCCAGAAGGGCTACGAGAAGTTCGAGTTCAACATCGAGGACTGCCGCAAGTTCATCGACTTCTACAAGCAGAGCATCTCCAAGCACCCGGAGTGGAAGGACTTCGGCTTCCGCTTCAGCGACACCCAGCGCTACAACTCCATCGACGAGTTCTACCGCGAGGTCGAGAACCAGGGCTACAAGCTGACCTTCGAGAACATCTCGGAGAGCTACATCGACAGCGTCGTGAACCAGGGCAAGCTGTACCTGTTCCAGATTTACAACAAGGACTTCAGCGCCTACTCCAAGGGCCGCCCTAACCTGCACACCCTGTACTGGAAGGCCCTGTTCGACGAGCGCAACCTCCAGGACGTCGTGTACAAGCTGAACGGCGAGGCCGAGCTGTTCTACCGCAAGCAGTCCATCCCGAAGAAGATCACCCACCCCGCCAAGGAGGCCATCGCCAACAAGAACAAGGACAACCCGAAGAAGGAGTCGGTGTTCGAGTACGACCTGATCAAGGACAAGCGCTTCACCGAGGACAAGTTCTTCTTCCACTGCCCCATCACCATCAACTTCAAGTCCTCGGGCGCCAACAAGTTCAACGACGAGATCAACCTGCTGCTGAAGGAGAAGGCCAACGACGTCCACATCCTGTCCATCGACCGCGGCGAGCGCCACCTGGCTTACTACACCCTGGTGGACGGCAAGGGCAACATCATCAAGCAGGACACCTTCAACATCATCGGCAACGACCGCATGAAGACCAACTACCACGACAAGCTGGCCGCTATCGAGAAGGACCGCGACTCCGCCCGCAAGGACTGGAAGAAGATCAACAACATCAAGGAGATGAAGGAGGGCTACCTGTCGCAGGTCGTGCACGAGATCGCCAAGCTGGTCATCGAGTACAACGCCATCGTCGTGTTCGAGGACCTGAACTTCGGCTTCAAGCGCGGTCGCTTCAAGGTCGAGAAGCAGGTGTACCAGAAGCTGGAGAAGATGCTGATCGAGAAGCTGAACTACCTGGTCTTCAAGGACAACGAGTTCGACAAGACCGGCGGCGTGCTGCGCGCTTACCAGCTGACCGCCCCGTTCGAGACCTTCAAGAAGATGGGCAAGCAGACCGGCATCATCTACTACGTCCCTGCCGGCTTCACCAGCAAGATTTGCCCCGTCACCGGCTTCGTGAACCAGCTGTACCCCAAGTACGAGTCCGTGTCGAAGAGCCAGGAGTTCTTCAGCAAGTTCGACAAGATTTGCTACAACCTGGACAAGGGCTACTTCGAGTTCTCCTTCGACTACAAGAACTTCGGCGACAAGGCCGCCAAGGGCAAGTGGACCATCGCCTCGTTCGGCAGCCGCCTGATCAACTTCCGCAACAGCGACAAGAACCACAACTGGGACACCCGCGAGGTCTACCCGACCAAGGAGCTGGAGAAGCTGCTGAAGGACTACTCCATCGAGTACGGCCACGGCGAGTGCATCAAGGCCGCCATCTGCGGCGAGAGCGACAAGAAGTTCTTCGCCAAGCTGACCTCCGTGCTGAACACCATCCTCCAGATGCGCAACAGCAAGACCGGCACCGAGCTGGACTACCTGATCTCCCCCGTCGCCGACGTGAACGGCAACTTCTTCGACTCCCGCCAGGCTCCTAAGAACATGCCTCAGGACGCTGACGCTAACGGCGCTTACCACATCGGCCTGAAGGGCCTGATGCTGCTGGGCCGCATCAAGAACAACCAGGAGGGCAAGAAGCTGAACCTGGTCATCAAGAACGAGGAGTACTTCGAGTTCGTGCAGAACCGCAACAACAGCCGCGCCGAC**CCGAAGAAGAAGCGCAAGGTG**TAG |

| **eGFP+ SV40 NLS (744 bp)** |
| --- |
| ATGGGGTACCATGGTCAGCAAGGGCGAGGAGCTGTTCACCGGCGTCGTGCCGATCCTGGTCGAGCTGGACGGCGACGTGAACGGCCACAAGTTCAGCGTCTCCGGCGAGGGCGAGGGCGACGCCACCTACGGCAAGCTGACCCTGAAGTTCATCTGCACCACCGGCAAGCTGCCGGTCCCCTGGCCTACCCTGGTGACCACCCTGACCTACGGCGTGCAGTGCTTCAGCCGCTACCCCGACCACATGAAGCAGCACGACTTCTTCAAGTCCGCTATGCCTGAGGGCTACGTCCAGGAGCGCACCATCTTCTTCAAGGACGACGGCAACTACAAGACCCGCGCCGAGGTCAAGTTCGAGGGCGACACCCTGGTGAACCGCATCGAGCTGAAGGGCATCGACTTCAAGGAGGACGGCAACATCCTGGGCCACAAGCTGGAGTACAACTACAACTCCCACAACGTCTACATCATGGCCGACAAGCAGAAGAACGGCATCAAGGTGAACTTCAAGATCCGCCACAACATCGAGGACGGCTCGGTCCAGCTGGCCGACCACTACCAGCAGAACACCCCTATCGGCGACGGCCCTGTGCTGCTGCCGGACAACCACTACCTGAGCACCCAGAGCGCCCTGTCCAAGGACCCGAACGAGAAGCGCGACCACATGGTCCTGCTGGAGTTCGTGACCGCCGCCGGCATCACCCTGGGCATGGACGAGCTGTACAAGCCT**CCGAAGAAGAAGCGCAAAGTG**TAG |

| **pCRISPR01 expression vector:**  **AMA1, Kan R, TEF1-P, TEF-TT, MCS** |
| --- |
| AGGTAAATCCCCACTACCGCATTAAGACCTCAGCGCGGCCGCAAATTTAAATAAAATGAAGTGAAGTTCCTATACTTTCTAGAGAATAGGAACTTCTATAGTGAGTCGAATAAGGGCGACACAAAATTTATTCTAAATGCATAATAAATACTGATAACATCTTATAGTTTGTATTATATTTTGTATTATCGTTGACATGTATAATTTTGATATCAAAAACTGATTTTCCCTTTATTATTTTCGAGATTTATTTTCTTAATTCTCTTTAACAAACTAGAAATATTGTATATACAAAAAATCATAAATAATAGATGAATAGTTTAATTATAGGTGTTCATCAATCAAAAAAGCAACGTATCTTATTTAAAGTGCGTTGCTTTTTTCTCATTTATAAGGTTAAATAATTCTCATATATCAAGCAAAGTGACAGGCGCCCTTAAATATTCTGACAAATGCTCTTTCCCTAAACTCCCCCCATAAAAAAACCCGCCGAAGCGGGTTTTTACGTTATTTGCGGATTAACGATTACTCGTTATCAGAACCGCCCAGGGGGCCCGAGCTTAAGACTGGCCGTCGTTTTACAACACAGAAAGAGTTTGTAGAAACGCAAAAAGGCCATCCGTCAGGGGCCTTCTGCTTAGTTTGATGCCTGGCAGTTCCCTACTCTCGCCTTCCGCTTCCTCGCTCACTGACTCGCTGCGCTCGGTCGTTCGGCTGCGGCGAGCGGTATCAGCTCACTCAAAGGCGGTAATACGGTTATCCACAGAATCAGGGGATAACGCAGGAAAGAACATGTGAGCAAAAGGCCAGCAAAAGGCCAGGAACCGTAAAAAGGCCGCGTTGCTGGCGTTTTTCCATAGGCTCCGCCCCCCTGACGAGCATCACAAAAATCGACGCTCAAGTCAGAGGTGGCGAAACCCGACAGGACTATAAAGATACCAGGCGTTTCCCCCTGGAAGCTCCCTCGTGCGCTCTCCTGTTCCGACCCTGCCGCTTACCGGATACCTGTCCGCCTTTCTCCCTTCGGGAAGCGTGGCGCTTTCTCATAGCTCACGCTGTAGGTATCTCAGTTCGGTGTAGGTCGTTCGCTCCAAGCTGGGCTGTGTGCACGAACCCCCCGTTCAGCCCGACCGCTGCGCCTTATCCGGTAACTATCGTCTTGAGTCCAACCCGGTAAGACACGACTTATCGCCACTGGCAGCAGCCACTGGTAACAGGATTAGCAGAGCGAGGTATGTAGGCGGTGCTACAGAGTTCTTGAAGTGGTGGGCTAACTACGGCTACACTAGAAGAACAGTATTTGGTATCTGCGCTCTGCTGAAGCCAGTTACCTTCGGAAAAAGAGTTGGTAGCTCTTGATCCGGCAAACAAACCACCGCTGGTAGCGGTGGTTTTTTTGTTTGCAAGCAGCAGATTACGCGCAGAAAAAAAGGATCTCAAGAAGATCCTTTGATCTTTTCTACGGGGTCTGACGCTCAGTGGAACGACGCGCGCGTAACTCACGTTAAGGGATTTTGGTCATGAGCTTGCGCCGTCCCGTCAAGTCAGCGTAATGCTCTGCTTACCAATGCTTAATCAGTGAGGCACCTATCTCAGCGATCTGTCTATTTCGTTCATCCATAGTTGCCTGACTCCCCGTCGTGTAGATAACTACGATACGGGAGGGCTTACCATCTGGCCCCAGCGCTGCGATGATACCGCGAGAACCACGCTCACCGGCTCCGGATTTATCAGCAATAAACCAGCCAGCCGGAAGGGCCGAGCGCAGAAGTGGTCCTGCAACTTTATCCGCCTCCATCCAGTCTATTAATTGTTGCCGGGAAGCTAGAGTAAGTAGTTCGCCAGTTAATAGTTTGCGCAACGTTGTTGCCATCGCTACAGGCATCGTGGTGTCACGCTCGTCGTTTGGTATGGCTTCATTCAGCTCCGGTTCCCAACGATCAAGGCGAGTTACATGATCCCCCATGTTGTGCAAAAAAGCGGTTAGCTCCTTCGGTCCTCCGATCGTTGTCAGAAGTAAGTTGGCCGCAGTGTTATCACTCATGGTTATGGCAGCGCTACATAATTCTCTTACTGTCATGCCATCCGTAAGATGCTTTTCTGTGACTGGTGAGTACTCAACCAAGTCATTCTGAGAATAGTGTATGCGGCGACCGAGTTGCTCTTGCCCGGCGTCAATACGGGATAATACCGCGCCACATAGCAGAACTTTAAAAGTGCTCATCATTGGAAAACGTTCTTCGGGGCGAAAACTCTCAAGGATCTTACCGCTGTTGAGATCCAGTTCGATGTAACCCACTCGTGCACCCAACTGATCTTCAGCATCTTTTACTTTCACCAGCGTTTCTGGGTGAGCAAAAACAGGAAGGCAAAATGCCGCAAAAAAGGGAATAAGGGCGACACGGAAATGTTGAATACTCATATTCTTCCTTTTTCAATATTATTGAAGCATTTATCAGGGTTATTGTCTCATGAGCGGATACATATTTGAATGTATTTAGAAAAATAAACAAATAGGGGTCAGTGTTACAACCAATTAACCAATTCTGAACATTATCGCGAGCCCATTTATACCTGAATATGGCTCATAACACCCCTTGTTTGCCTGGCGGCAGTAGCGCGGTGGTCCCACCTGACCCCATGCCGAACTCAGAAGTGAAACGCCGTAGCGCCGATGGTAGTGTGGGGACTCCCCATGCGAGAGTAGGGAACTGCCAGGCATCAAATAAAACGAAAGGCTCAGTCGAAAGACTGGGCCTTTCGCCCGGGCTAATTATGGGGTGTCGCCCTTATTCGACTCTATAGTGAAGTTCCTATTCTCTAGAAAGTATAGGAACTTCTGAAGTGGGGATTTAAATGCGGCCGCGCTGAGGGTTTAATCGACGAAGCAGCTGACGGCCAGTGCCAAGCTTAACGCGTACCGGGCCCAGTATATGTTCCGCAGATGACTGGAGCTCTGCCATACGTGCCCTCTCAAGCACCATTTGTTCCATCTACAGAGACTAGTCACCAACTAGTCTATCAAGACTCACAGGGTACATTGCTGAGACCAACTGACCAGAGGCAGGGTAGCGGATTGACGGCTCCATCTCCTTCACTTACAAGGTCTATTGAAAGCCCTTTAGCATCACCAAGCGGAGAATAGATTGTTAAGCTTATTTTTTGTATACTGTTTTGTGATAGCACGAAGTTTTTCCACGGTATCTTGTTAAAAATATATATTTGTGGCGGGCTTACCTACATCAAATTAATAAGAGACTAATTATAAACTAAACACACAAGCAAGCTACTTTAGGGTAAAAGTTTATAAATGCTTTTGACGTATAAACGTTGCTTGTATTTATTATTACAATTAAAGGTGGATAGAAAACCTAGAGACTAGTTAGAAACTAATCTCAGGTTTGCGTTAAACTAAATCAGAGCCCGAGAGGTTAACAGAACCTAGAAGGGGACTAGATATCCGGGTAGGGAAACAAAAAAAAAAAACAAGACAGCCACATATTAGGGAGACTAGTTAGAAGCTAGTTCCAGGACTAGGAAAATAAAAGACAATGATACCACAGTCTAGTTGACAACTAGATAGATTCTAGATTGAGGCCAAAGTCTCTGAGATCCAGGTTAGTTGCAACTAATACTAGTTAGTATCTAGTCTCCTATAACTCTGAAGCTAGAATAACTTACTACTATTATCCTCACCACTGTTCAGCTGCGCAAACGGAGTGATTGCAAGGTGTTCAGAGACTAGTTATTGACTAGTCAGTGACTAGCAATAACTAACAAGGTATTAACCTACCATGTCTGCCATCACCCTGCACTTCCTCGGGCTCAGCAGCCTTTTCCTCCTCATTTTCATGCTCATTTTCCTTGTTTAAGACTGTGACTAGTCAAAGACTAGTCCAGAACCACAAAGGAGAAATGTCTTACCACTTTCTTCATTGCTTGTCTCTTTTGCATTATCCATGTCTGCAACTAGTTAGAGTCTAGTTAGTGACTAGTCCGACGAGGACTTGCTTGTCTCCGGATTGTTGGAGGAACTCTCCAGGGCCTCAAGATCCACAACAGAGCCTTCTAGAAGACTGGTCAATAACTAGTTGGTCTTTGTCTGAGTCTGACTTACGAGGTTGCATACTCGCTCCCTTTGCCTCGTCAATCGATGAGAAAAAGCGCCAAAACTCGCAATATGGCTTTGAACCACACGGTGCTGAGACTAGTTAGAATCTAGTCCCAAACTAGCTTGGATAGCTTACCTTTGCCCTTTGCGTTGCGACAGGTCTTGCAGGGTATGGTTCCTTTCTCACCAGCTGATTTAGCTGCCTTGCTACCCTCACGGCGGATCTGCATAAAGAGTGGCTAGAGGTTATAAATTAGCACTGATCCTAGGTACGGGGCTGAATGTAACTTGCCYTTCCTTTCTCATCGCGCGGCAAGACAGGCTTGCTCAAATTCCTACCAGTCACAGGGGTATGCACGGCGTACGGACCACTTGAACTAGTCACAGATTAGTTAGCAACTAGTCTGCATTGAATGGCTGTACTTACGGGCCCTCGCCATTGTCCTGATCATTTCCAGCTTCACCCTCGTTGCTGCAAAGTAGTTAGTGACTAGTCAAGGACTAGTTGAAATGGGAGAAGAAACTCACGAATTCTCGACACCCTTAGTATTGTGGTCCTTGGACTTGGTGCTGCTATATATTAGCTAATACACTAGTTAGACTCACAGAAACTTACGCAGCTCGCTTGCGCTTCTTGGTAGGAGTCGGGGTTGGGAGAACAGTGCCTTCAAACAAGCCTTCATACCATGCTACTTGACTAGTCAGGGACTAGTCACCAAGTAATCTAGATAGGACTTGCCTTTGGCCTCCATCAGTTCCTTCATAGTGGGAGGTCCATTGTGCAATGTAAACTCCATGCCGTGGGAGTTCTTGTCCTTCAAGTGCTTGACCAATATGTTTCTGTTGGCAGAGGGAACCTGTCAACTAGTTAATAACTAGTCAGAAACTAGTATAGCAGTAGACTCACTGTACGCTTGAGGCATCCCTTCACTCGGCAGTAGACTTCATATGGATGGATATCAGGCACGCCATTGTCGTCCTGTGGACTAGTCAGTAACTAGGCTTAAAGCTAGTCGGGTCGGCTTACTATCTTGAAATCCGGCAGCGTAAGCTCCCCGTCCTTAACTGCCTCGAGATAGTGACAGTACTCTGGGGACTTTCGGAGATCGTTATCGCGAATGCTCGGCATACTAATCGTTGACTAGTCTTGGACTAGTCCCGAGCAAAAAGGATTGGAGGAGGAGGAGGAAGGTGAGAGTGAGACAAAGAGCGAAATAAGAGCTTCAAAGGCTATCTCTAAGCAGTATGAAGGTTAAGTATCTAGTTCTTGACTAGATTTAAAAGAGATTTCGACTAGTTATGTACCTGGAGTTTGGATATAGGAATGTGTTGTGGTAACGAAATGTAAGGGGGAGGAAAGAAAAAGTCGGTCAAGAGGTAACTCTAAGTCGGCCATTCCTTTTTGGGAGGCGCTAACCATAAACGGCATGGTCGACTTAGAGTTAGCTCAGGGAATTTAGGGAGTTATCTGCGACCACCGAGGAACGGCGGAATGCCAAAGAATCCCGATGGAGCTCTAGCTGGCGGTTGACAACCCCACCTTTTGGCGTTTCTGCGGCGTTGCAGGCGGGACTGGATACTTCGTAGAACCAGAAAGGCAAGGCAGAACGCGCTCAGCAAGAGTGTTGGAAGTGATAGCATGATGTGCCTTGTTAACTAGGTCAAAATCTGCAGTATGCTTGATGTTATCCGAAGTGTGAGAGAGGAAGGTCCAAACATACACGATTGGGAGAGGGCCTAGGTATAAGAGTTTTTGAGTAGAACGCATGTGAGCCCAGCCATCTCGAGGAGATTAAACACGGGCCGGCATTTGATGGCTATGTTAGTACCCCAATGGAAACGGTGAGAGTCCAGTGGTCGCAGATAACTCCCTAAATTCCCTGAGCTAACTCTAAGTCGACCATGCCGTTTATGGTTAGCGCCTCCCAAAAAGGAATGGCCGACTTAGAGTTACCTCTTGACCGACTTTTTCTTTCCTCCCCCTTACATTTCGTTACCACAACACATTCCTATATCCAAACTCCAGGTACATAACTAGTCGAAATCTCTTTTAAATCTAGTCAAGAACTAGATACTTAACCTTCATACTGCTTAGAGATAGCCTTTGAAGCTCTTATTTCGCTCTTTGTCTCACTCTCACCTTCCTCCTCCTCCTCCAATCCTTTTTGCTCGGGACTAGTCCAAGACTAGTCAACGATTAGTATGCCGAGCATTCGCGATAACGATCTCCGAAAGTCCCCAGAGTACTGTCACTATCTCGAGGCAGTTAAGGACGGGGAGCTTACGCTGCCGGATTTCAAGATAGTAAGCCGACCCGACTAGCTTTAAGCCTAGTTACTGACTAGTCCACAGGACGACAATGGCGTGCCTGATATCCATCCATATGAAGTCTACTGCCGAGTGAAGGGATGCCTCAAGCGTACAGTGAGTCTACTGCTATACTAGTTTCTGACTAGTTATTAACTAGTTGACAGGTTCCCTCTGCCAACAGAAACATATTGGTCAAGCACTTGAAGGACAAGAACTCCCACGGCATGGAGTTTACATTGCACAATGGACCTCCCACTATGAAGGAACTGATGGAGGCCAAAGGCAAGTCCTATCTAGATTACTTGGTGACTAGTCCCTGACTAGTCAAGTAGCATGGTATGAAGGCTTGTTTGAAGGCACTGTTCTCCCAACCCCGACTCCTACCAAGAAGCGCAAGCGAGCTGCGTAAGTTTCTGTGAGTCTAACTAGTGTATTAGCTAATATATAGCAGCACCAAGTCCAAGGACCACAATACTAAGGGTGTCGAGAATTCGTGAGTTTCTTCTCCCATTTCAACTAGTCCTTGACTAGTCACTAACTACTTTGCAGCAACGAGGGTGAAGCTGGAAATGATCAGGACAATGGCGAGGGCCCGTAAGTACAGCCATTCAATGCAGACTAGTTGCTAACTAATCTGTGACTAGTTCAAGTGGTCCGTACGCCGTGCATACCCCTGTGACTGGTAGGAATTTGAGCAAGCCTGTCTTGCCGCGCGATGAGAAAGGAARGGCAAGTTACATTCAGCCCCGTACCTAGGATCAGTGCTAATTTATAACCTCTAGCCACTCTTTATGCAGATCCGCCGTGAGGGTAGCAAGGCAGCTAAATCAGCTGGTGAGAAAGGAACCATACCCTGCAAGACCTGTCGCAACGCAAAGGGCAAAGGTAAGCTATCCAAGCTAGTTTGGGACTAGATTCTAACTAGTCTCAGCACCGTGTGGTTCAAAGCCATATTGCGAGTTTTGGCGCTTTTTCTCATCGATTGACGAGGCAAAGGGAGCGAGTATGCAACCTCGTAAGTCAGACTCAGACAAAGACCAACTAGTTATTGACCAGTCTTCTAGAAGGCTCTGTTGTGGATCTTGAGGCCCTGGAGAGTTCCTCCAACAATCCGGAGACAAGCAAGTCCTCGTCGGACTAGTCACTAACTAGACTCTAACTAGTTGCAGACATGGATAATGCAAAAGAGACAAGCAATGAAGAAAGTGGTAAGACATTTCTCCTTTGTGGTTCTGGACTAGTCTTTGACTAGTCACAGTCTTAAACAAGGAAAATGAGCATGAAAATGAGGAGGAAAAGGCTGCTGAGCCCGAGGAAGTGCAGGGTGATGGCAGACATGGTAGGTTAATACCTTGTTAGTTATTGCTAGTCACTGACTAGTCAATAACTAGTCTCTGAACACCTTGCAATCACTCCGTTTGCGCAGCTGAACAGTGGTGAGGATAATAGTAGTAAGTTATTCTAGCTTCAGAGTTATAGGAGACTAGATACTAACTAGTATTAGTTGCAACTAACCTGGATCTCAGAGACTTTGGCCTCAATCTAGAATCTATCTAGTTGTCAACTAGACTGTGGTATCATTGTCTTTTATTTTCCTAGTCCTGGAACTAGCTTCTAACTAGTCTCCCTAATATGTGGCTGTCTTGTTTTTTTTTTTTTTGTTTCCCTACCCGGATATCTAGTCCCCTTCTAGGTTCTGTTAACCTCTCGGGCTCTGATTTAGTTTAACGCAAACCTGAGATTAGTTTCTAACTAGTCTCTAGGTTTTCTATCCACCTTTAATTGTAATAATAAATACAAGCAACGTTTATACGTCAAAAGCATTTATAAACTTTTACCCTAAAGTAGCTTGCTTGTGTGTTTAGTTTATAATTAGTCTCTTATTAATTTGATGTAGGTAAGCCCGCCACAAATATATATTTTTAACAAGATACCGTGGAAAAACTTCGTGCTATCACAAAACAGTATACAAAAAATAAGCTTAACAATCTATTCTCCGCTTGGTGATGCTAAAGGGCTTTCAATAGACCTTGTAAGTGAAGGAGATGGAGCCGTCAATCCGCTACCCTGCCTCTGGTCAGTTGGTCTCAGCAATGTACCCTGTGAGTCTTGATAGACTAGTTGGTGACTAGTCTCTGTAGATGGAACAAATGGTGCTTGAGAGGGCACGTATGGCAGAGCTCCAGTCATCTGCGGAACATATACTGGGCCCGGGAAGATCCGATATCGCCGTGGCGGCCGCTCTAGAACTAGTGGATCGATCCCCAATTCGCCCTATAGTGAGTCGTATTACAATTCACTGGCCGTCGTTTTACAACGTCGTGACTGGGAAAACCCTGGCGTTACCCAACTTAATCGCCTTGCAGCACATCCCCCTTTCGCCAGCTGGCGTAATAGCGAAGAGGCCCGCACCGATCGCCCTTCCCAACAGTTGCGCAGCCTGAATGGCGAATGAGCTTGCGCCGTCCCGTCAAGTCAGCGTAATGCTCTGCCAGTGTTACAACCAATTAACCAATTCTGATTAGAAAAACTCATCGAGCATCAAATGAAACTGCAATTTATTCATATCAGGATTATCAATACCATATTTTTGAAAAAGCCGTTTCTGTAATGAAGGAGAAAACTCACCGAGGCAGTTCCATAGGATGGCAAGATCCTGGTATCGGTCTGCGATTCCGACTCGTCCAACATCAATACAACCTATTAATTTCCCCTCGTCAAAAATAAGGTTATCAAGTGAGAAATCACCATGAGTGACGACTGAATCCGGTGAGAATGGCAAAAGTTTATGCATTTCTTTCCAGACTTGTTCAACAGGCCAGCCATTACGCTCGTCATCAAAATCACTCGCATCAACCAAACCGTTATTCATTCGTGATTGCGCCTGAGCGAGACGAAATACGCGATCGCTGTTAAAAGGACAATTACAAACAGGAATCGAATGCAACCGGCGCAGGAACACTGCCAGCGCATCAACAATATTTTCACCTGAATCAGGATATTCTTCTAATACCTGGAATGCTGTTTTTCCGGGGATCGCAGTGGTGAGTAACCATGCATCATCAGGAGTACGGATAAAATGCTTGATGGTCGGAAGAGGCATAAATTCCGTCAGCCAGTTTAGTCTGACCATCTCATCTGTAACATCATTGGCAACGCTACCTTTGCCATGTTTCAGAAACAACTCTGGCGCATCGGGCTTCCCATACAAGCGATAGATTGTCGCACCTGATTGCCCGACATTATCGCGAGCCCATTTATACCCATATAAATCAGCATCCATGTTGGAATTTAATCGCGGCCTCGACGTTTCCCGTTGAATATGGCTCATAACACCCCTTGTATTACTGTTTATGTAAGCAGACAGTTTTATTGTTCATGATGATATATTTTTATCTTGTGCAATGTAACATCAGAGATTTTGAGACACAACGTGGCTTTCCCCCCCCCCCCTCGACTCTAGGATAATCTCATGACCAAAATCCCTTAACGTGAGTTTTCGTTCCACTGAGCGTCAGACCCCTTAATAAGATGATCTTCAGATCTCATGGTCATAGCTGTTTCCGCTGAGGGTTTAATTAAGACCTCAGCCGAGACAGCAGAATCACCGCCCAAGTTAAGCCTTTGTGCTGATCATGCTCTCGAACGGGCCAAGTTCGGGAAAAGCAAAGGAGCGTTTAGTGAGGGGCAATTTGACTCACCTCCCAGGCAACAGATGAGGGGGGCAAAAAGAAAGAAATTTTCGTGAGTCAATATGGATTCCGAGCATCATTTTCTTGCGGTCTATCTTGCTACGTATGTTGATCTTGACGCTGTGGATCAAGCAACGCCACTCGCTCGCTCCATCGCAGGCTGGTCGCAGACAAATTAAAAGGCGGCAAACTCGTACAGCCGCGGGGTTGTCCGCTGCAAAGTACAGAGTGATAAAAGCCGCCATGCGACCATCAACGCGTTGATGCCCAGCTTTTTCGATCCGAGAATCCACCGTAGAGGCGATAGCAAGTAAAGAAAAGCTAAACAAAAAAAAATTTCTGCCCCTAAGCCATGAAAACGAGATGGGGTGGAGCAGAACCAAGGAAAGAGTCGCGCTGGGCTGCCGTTCCGGAAGGTGTTGTAAAGGCTCGACGCCCAAGGTGGGAGTCTAGGAGAAGAATTTGCATCGGGAGTGGGGCGGGTTACCCCTCCATATCCAATGACAGATATCTACCAGCCAAGGGTTTGAGCCCGCCCGCTTAGTCGTCGTCCTCGCTTGCCCCTCCATAAAAGGATTTCCCCTCCCCCTCCCACAAAATTTTCTTTCCCTTCCTCTCCTTGTCCGCTTCAGTACGTATATCTTCCCTTCCCTCGCTTCTCTCCTCCATCCTTCTTTCATCCATCTCCTGCTAACTTCTCTGCTCAGCACCTCTACGCATTACTAGCCGTAGTATCTGAGCACTTCTCCCTTTTATATTCCACAAAACATAACACAACCTTCA**CCATGGGGTACCGTTTAAAC**GCGGACATTCGATTTATGCCGTTATGACTTCCTTAAAAAAGCCTTTACGAATGAAAGAAATGGAATTAGACTTGTTATGTAGTTGATTCTACAATGGATTATGATTCCTGAACTTCAAATCCGCTGTTCATTATTAATCTCAGCTCTTCCCGTAAAGCCAATGTTGAAACTATTCGTAAATGTACCTCGTTTTGCGTGTACCTTGCTTATCACGTGATATTACATGACCTGGACAGAGTTCTGCGCGAAAGTCATAACGTAAATCCCGGGCGGTAGGTGCGTCCCGGGCGGAAGGTAGTTTTCTCGTCCACCCCAACGCGTTTATCAACCTCAACTTTCAACAACCATCATGCCACCAAAAGCGCGTAAAACAAAGCGAGATTTGATTGAGCAAGAGGGCAGGATCCAATGCGCGATTCAAGACATTAAAAATGGAAAATTTCAAAAAATTGCGCCCGCAGCGCGTGCATACAAAATTCATCCCAATACAAGTGTAATGCTAGTGGAGGTCAACACATCAATGCCTATTTTGGTTTAGTCGTCCAGGCGGTGAGCACAAAATTTGTGTCGTTTGACAAGATGGTTCATTTAGGCAACTGGTCAGATCAGCCCCACTTGTAGCAGTAGCGGCGGCGCTCGAAGTGTGACTCTTATTAGCAGACAGGAACGAGGACATTATTATCATCTGCTGCTTGGTGCACGATAACTTGGTGCGTTTGTCAAGCAAGGTAAGTGGACGACCCGGTCATACCTTCTTAAGTTCGCCCTTCCTCCCTTTATTTCAGATTCAATCTGACTTACCTATTCTACCCAAGCATCCAAATGGCCAAGTTGACCAGTGCCGTTCCGGTGCTCACCGCGCGCGACGTCGCCGGAGCGGTCGAGTTCTGGACCGACCGGCTCGGGTTCTCCCGGGACTTCGTGGAGGACGACTTCGCCGGTGTGGTCCGGGACGACGTGACCCTGTTCATCAGCGCGGTCCAGGACCAGGTGGTGCCGGACAACACCCTGGCCTGGGTGTGGGTGCGCGGCCTGGACGAGCTGTACGCCGAGTGGTCGGAGGTCGTGTCCACGAACTTCCGGGACGCCTCCGGGCCGGCCATGACCGAGATCGGCGAGCAGCCGTGGGGGCGGGAGTTCGCCCTGCGCGACCCGGCCGGCAACTGCGTGCACTTCGTGGCCGAGGAGCAGGACTGACCGACGCCGACCAACACCGCCGGTCCGACGCGGCCCGACGGGTCCGAGGAGCTTGAGATCCACTTAACGTTACTGAAATCATCAAACAGCTTGACGAATCTGGATATAAGATCGTTGGTGTCGATGTCAGCTCCGGAGTTGAGACAAATGGTGTTCAGGATCTCGATAAGATACGTTCATTTGTCCAAGCAGCAAAGAGTGCCTTCTAGTGATTTAATAGCTCCATGTCAACAAGAATAAAACGCGTTTCGGGTTTACCTCTTCCAGATACAGCTCATCTGCAATGCATTAATGCATTGGACCTCGCAACCCTAGTACGCCCTTCAGGCTCCGGCGAAGCAGAAGAATAGCTTAGCAGAGTCTATTTTCATTTTCGGGAGACGAGATCAAGCAGATCAACGGTCGTCAAGAGACCTACGAGACTGAGGAATCCGCTCTTGGCTCCACGCGACTATATATTTGTCTCTAATTGTACTTTGACATGCTCCTCTTCTTTACTCTGATAGCTTGACTATGAAAATTCCGTCACCAGCCCCTGGGTTCGCAAAGATAATTGCACTGTTTCTTCCTTGAACTCTCAAGCCTACAGGACACACATTCATCGTAGGTATAAACCTCGAAAATCATTCCTACTAAGATGGGTATACAATAGTAACCATGCATGGTTGCCTAGTGAATGCTCCGTAACACCCAATACGCCGGCCGAAACTTTTTTACAACTCTCCTATGAGTCGTTTACCCAGAATGCACAGGTACACTTGTTTAG |
